# The Anti-Inflammatory Effects of a Yin Zhi Huang Soup in an Experimental Autoimmune Prostatitis Rat Model

**DOI:** 10.1155/2017/7312938

**Published:** 2017-12-21

**Authors:** Longsheng Deng, Xikui Zhang, Weikun Zhu, Taikun Lu, Jinchun Chen, Qiang Zou, Qizhong Zheng, Junying Chen, Changming Jiang, Guanyu Jin

**Affiliations:** ^1^Department of Andrology, Xiamen Hospital of Traditional Chinese Medicine, Xiamen, China; ^2^Department of Clinical Foundation of TCM, Fujian University of Traditional Chinese Medicine, Fuzhou, China

## Abstract

The present study aimed to investigate the therapeutic effects of the Chinese herbal medicine Yin Zhi Huang soup (YZS) in an experimental autoimmune prostatitis (EAP) rat model. In total, 48 rats were randomly divided into the following four groups (*n* = 12/group): saline group, pathological model group, Qianlietai group, and YZS group. We determined the average wet weight of the prostate tissue, the ratio of the wet weight of the prostate tissue to body weight, tumor necrosis factor-alpha (TNF-*α*) levels in the blood serum, the expression of inducible nitric oxide synthase (iNOS) in the rats' prostate tissues, and the pathological changes in the prostate tissue using light microscopy. YZS reduced the rats' prostate wet weight, the ratio of the prostate wet weight to body weight, and TNF-*α* levels in the blood serum and inhibited the expression of iNOS in the rats' prostate tissues (*P* < 0.05). Following YZS treatment, the pathological changes in the rats' prostates were improved compared with those in the model group (*P* < 0.05). Furthermore, YZS treatment reduced inflammatory changes in the prostate tissue. It also significantly suppressed proinflammatory cytokines, such as TNF-*α*, and chemokines, such as iNOS, in the rat model of EAP.

## 1. Introduction

Chronic prostatitis/chronic pelvic pain syndrome (CP/CPPS) is a male urogenital disease commonly observed in urological practice. The estimated global prevalence ranges from 2.2% to 9.7% [1]. Similar to diabetes mellitus, Crohn's disease, and myocardial infarction, CP/CPPS has unfavourable effects on physiological and psychological status and the quality of life of patients [2]. CP/CPPS causes pelvic pain (localized to the urethra, perineum, or prostate), inflammation of the prostate, urinary symptoms, and a variable degree of sexual dysfunction [3], although the aetiology and pathogenesis of CP/CPPS remain unclear. Medications (antimicrobials, anti-inflammatories, and alpha-blockers), pelvic floor training, phytotherapy, thermal therapies, and biofeedback have been shown to relieve CP/CPPS pain [3, 4]. However, most patients continue to feel uncomfortable, and thus CP/CPPS is a chronic disease that is difficult to treat. Inflammation has been extensively shown to play a significant role in CP/CPPS. Additionally, elevated levels of proinflammatory cytokines, such as TNF-*α*, and chemokines, such as iNOS, have been associated with CP/CPPS symptoms and diagnosis severity in patients [5, 6].

TNF-*α* is a very important proinflammatory cytokine, and its expression has been shown to rapidly increase in prostatic secretions of CP/CPPS patients. The increased TNF-*α* triggers iNOS to synthesize and release large amounts of nitric oxide (NO). These changes induce an obvious inflammatory response. Thus, efficiently controlling and reducing the TNF-*α* levels in blood serum and inhibiting iNOS expression in the prostate are key aims of CP/CPPS treatment [7, 8].

Researchers have recently established several animal models, including hormone-associated prostatitis models, immune-induced prostatitis models, and other miscellaneous prostatitis models. A reliable animal model is essential for revealing the aetiology and pathogenesis of CP/CPPS [9]. Li et al. [10] observed similar histological findings in spontaneously developed prostatitis and purified prostatic protein-induced EAP in Wistar rats. According to many studies, EAP in rats highly resembles CP/CPPS in humans [11, 12].

Herbal* Artemisia scoparia*,* Rheum palmatum* L., Fructus Gardeniae,* Artemisia anomala* S. Moore, Flos Chrysanthemi Indici, Semen Litchi, and* Taraxacum mongolicum* Hand.-Mazz are local Chinese herbs that are popular in Chinese medicine. These herbs can be used individually or in combination and have been demonstrated to exert various anti-inflammatory effects [13–18]. Furthermore, these herbs are the components of the new herbal formula called YZS. The aim of this study was to investigate the anti-inflammatory effects of YZS for the treatment of EAP in a rat model.

## 2. Materials and Methods

### 2.1. Preparation of the Herbal Formula YZS

YZS comprises the following seven Chinese herbs: herbal* Artemisia scoparia* (18 g),* Rheum palmatum* L. (6 g), Fructus Gardeniae (12 g),* Artemisia anomala* S. Moore (12 g), Flos Chrysanthemi Indici (15 g), Semen Litchi (12 g), and* Taraxacum mongolicum* Hand.-Mazz (6 g). The dosage is based on a book published by Shang Han Lun. All Chinese herbs were purchased from Fujian University of Traditional Chinese Medicine. All herbs were prepared using TCM decocting methods. The following steps were performed: (1) all herbs were added to a China pot with 400 ml water, (2) soaked for 30 minutes, and (3) boiled and then heated under a mild heat for approximately 20 minutes; (4) the liquid medicine was boiled to approximately 100 ml.

### 2.2. Animals

All animals (12-week-old male Wistar rats) were purchased from Shanghai SLAC Laboratory Animal Co., Ltd. The mice were kept in a pathogen-free animal room maintained at a constant temperature and humidity under a 12-hour light/dark cycle. All animal experiments followed the standard procedures of the Animal Care and Use Committee of the Fujian University of Traditional Chinese Medicine.

### 2.3. Preparation of Rat Purified Prostate Protein

Preparation of the rat model was performed according to the method described by Li et al. [10] and Jackson et al. [12]. The process was performed as follows: twelve male Wistar rats were sacrificed by cervical dislocation, the prostate tissue was removed, and the tissue samples were supplemented with several applications of 0.5% Triton X-100. The homogenate was homogenized using a glass homogenizer and centrifuged at 10000 r/min for 30 minutes to obtain the supernatant liquid. Bovine serum albumin was used as the standard protein. The protein content was determined using the biuret method with a 721 Spectrophotometer. Finally, the solution was diluted to 15 mg/ml with 0.1 mol/l of phosphate-buffered salt (PBS) buffer at pH 7.2.

### 2.4. Vaccination

Forty-eight rats were randomly divided into the following two groups: a normal saline group (12 rats) and a model group (36 rats). The model group was intraperitoneally injected with a diphtheria, acellular pertussis, and tetanus (DPT) combined vaccine, which was adsorbed to 0.5 ml. The animals received multisite intradermal injections of purified prostate protein and Freund's complete adjuvant (FCA) (1 ml; ratio of 1 : 1). The normal saline group was treated with an equal volume of normal saline by an intraperitoneal injection and multisite intradermal injections. After 6 weeks, the 36 rats of the model group were randomly divided into the following 3 subgroups (12 rats per group): a pathological model group, a Qianlietai group, and a YZS group. These groups were treated with 10 ml (kg/d) via oral gavage once daily for four weeks. The Qianlietai group and YZS group were given the corresponding medicine, and the normal saline group and pathological model group were given equal amounts of normal saline. TNF-*α* levels in the blood serum and the expression of iNOS in the rats' prostate tissues were measured after 4 weeks of treatment.

### 2.5. Materials

A TNF-*α* kit (Shanghai Jiang Lai Biological Technology Co., Ltd.), an iNOS kit (Wuhan Boster Biological Technology Ltd.), and Qianlietai tablets (Gansu Hexi Pharmaceutical Co., Ltd., batch number: 041205) were used. The 6.6 g/100 ml liquid consisted of tablet powder and saline.

### 2.6. Test Method

We detected TNF-*α* content in the rat serum by the absorbance (Value A) of the blood sample at a wavelength of 490 nm minus the absorbance of a blank. According to Value A of the sample, the corresponding TNF-*α* content can be calculated using a calibration curve.

iNOS immunohistochemistry detection was performed using a double-blind method, and pale brown particles in the cytoplasm of the prostate tissue were observed as the positive control. The detection was graded according to the staining intensity and the number of positive cells. Three images were randomly observed for each section under a microscope. The selected 100 cells were counted as the percentage of cells in each position under a high-power microscope (×400). Percentages <10% were scored as 0 points, 10~25% were scored as 1 point, 26~50% were scored as 2 points, and >51% were scored as 3 points. The tinting strength was scored as follows: no colouring or colouring similar to the background was scored as 0 points; light yellow colouring or colouring slightly higher than the background was scored as 1 point; yellow colouring or colouring clearly higher than the background was scored as 2 points; and remarkable colouring or deep brown was scored as 3 points. The two scores were combined as follows: 0~1 points indicated negative staining, 2 points indicated positive staining, and >3 points indicated strong positive staining.

### 2.7. Histological Analyses

The prostate tissue was collected after the rats were sacrificed and then fixed with 10% formalin for 12 hours. 2 mm thick sections were obtained via a large vertical urethral cut on the surface. Then, 75~100% ethanol was used to gradually dehydrate the sample, followed by treatment with xylene transparent solution, dipping in wax, embedding in paraffin, serial sectioning at 3 *μ*m, and staining with haematoxylin and eosin.

The prostate tissue was observed, and the pathological changes and morphology of the rat prostate tissue were photographed throughout the experiment.

### 2.8. Pathological Grading Standards in the Rat EAP Model

*Grade 0*. There was no inflammatory cell infiltration present in the prostate stroma and uniform pink secretions were observed in the glandular cavity.

*Grade 1*. A small amount of inflammatory cell infiltration was observed in the stroma with no change in the glandular secretions. 

*Grade 2*. A large amount of inflammatory cell infiltration was observed in the stroma with slight secretion in the glandular lumen. 

*Grade 3*. A large amount of inflammatory cell infiltration was observed in the stroma, and secretion in the glandular cavity was obviously reduced or was absent.

In the statistical analyses to compare the groups, level 0 was 0, level 1 was 2, level 2 was 4, and level 3 was 6.

### 2.9. Statistical Analysis

The data are represented as $\overset{-}{x}\pm s$, and SPSS 13.0 software for Windows was used for the statistical analysis. The measurement data were analysed using a single-factor analysis of variance, and the count data were analysed using *X*^2^ test. *P* < 0.05 was considered statistically significant.

## 3. Results

### 3.1. Prostate Tissue Wet Weight and Comparison of Prostate Tissue Wet Weight to Body Weight

**Figure 1:**
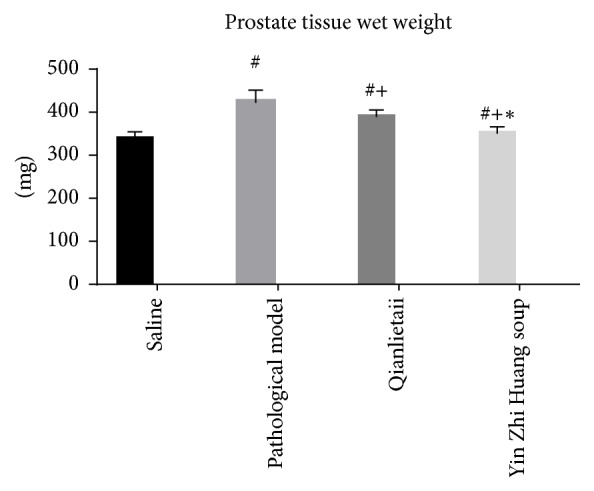
Prostate tissue wet weight. # indicates comparison of # and saline group, *P* < 0.05; + indicates comparison of + and pathological model group, *P* < 0.05; *∗* indicates comparison of *∗* and Qianlietai group, *P* < 0.05.

**Figure 2:**
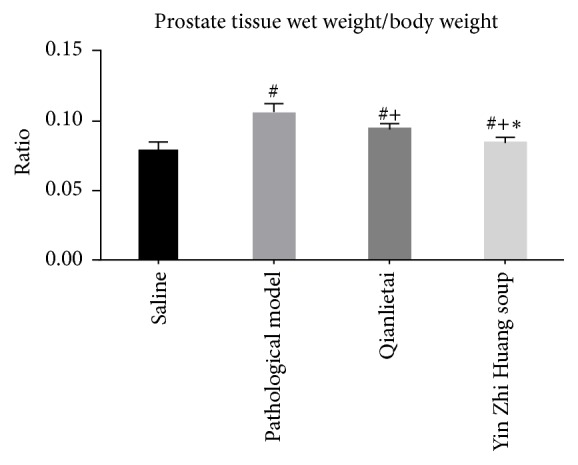
Ratio of the prostate tissue wet weight to the body weight. # indicates comparison of # and saline group, *P* < 0.05; + indicates comparison of + and pathological model group, *P* < 0.05; *∗* indicates comparison of *∗* and Qianlietai group, *P* < 0.05.

Significant differences were observed in the changes between the pathological model group and the saline group. The prostate wet weight and the ratio of the prostate wet weight to the body weight clearly increased. These values decreased in the Qianlietai group and the YZS group compared with the pathological model group, but a more obvious decrease was observed in the YZS group (see Figures 1 and 2).

### 3.2. Effect of the YZS Treatment on TNF-*α* Content

**Figure 3:**
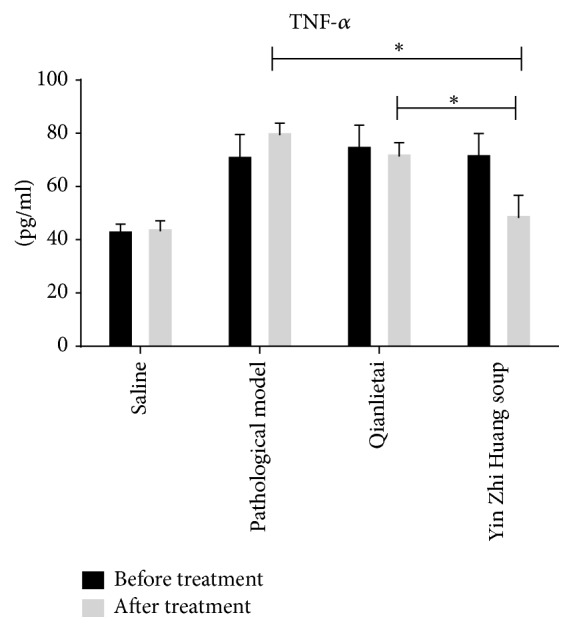
Effects of different therapies on experimental autoimmune prostatitis as evaluated by TNF-*α*.* Note*. A significant difference was observed between the two groups, *P* < 0.05. *∗* indicates the comparison between the two groups.

The results of TNF-*α* are shown in Figure 3. The content of TNF-*α* in the pathological model group was higher than that in the saline group. TNF-*α* content did not obviously change after treatment with the Qianlietai tablets, although the YZS group exhibited significantly reduced TNF-*α* levels in the blood serum (Figure 3).

### 3.3. Effect of YZS Treatment on iNOS Expression

**Figure 4:**
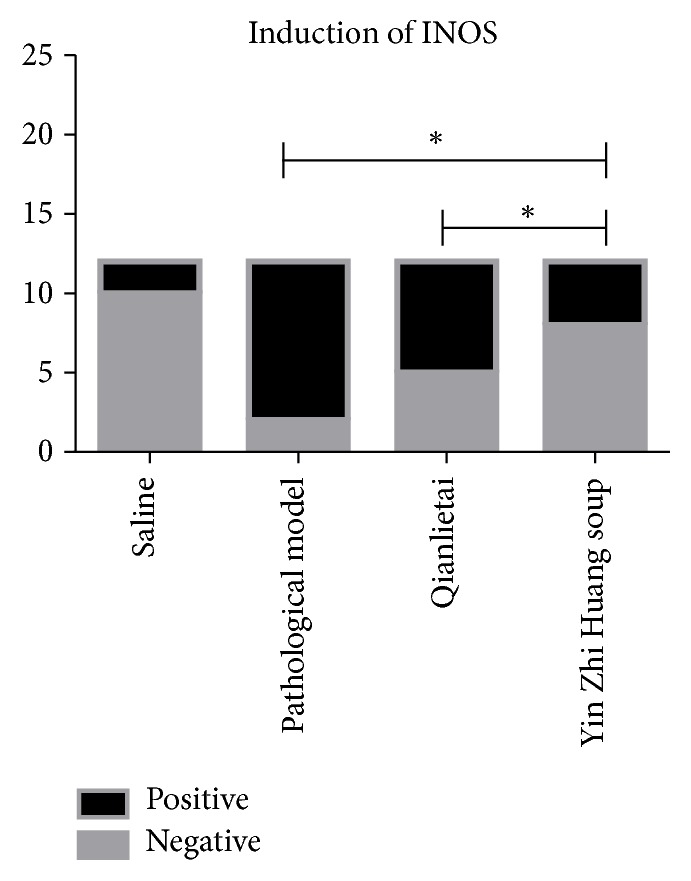
Effects of different therapies on experimental autoimmune prostatitis as evaluated by iNOS expression. *∗* indicates the comparison between the two groups.

According to the analysis of iNOS expression in the rat prostate tissue, (1) iNOS was overexpressed in the prostate tissue during EAP development and occurrence; (2) iNOS expression in the Qianlietai group was higher than that in the pathological group, but no significant differences were observed between the two groups (*P* > 0.05); and (3) the YZS group exhibited greater inhibition of iNOS expression than the Qianlietai group (*P* < 0.05) (Figure 4).

### 3.4. Histopathology after the YZS Treatment

**Figure 5:**
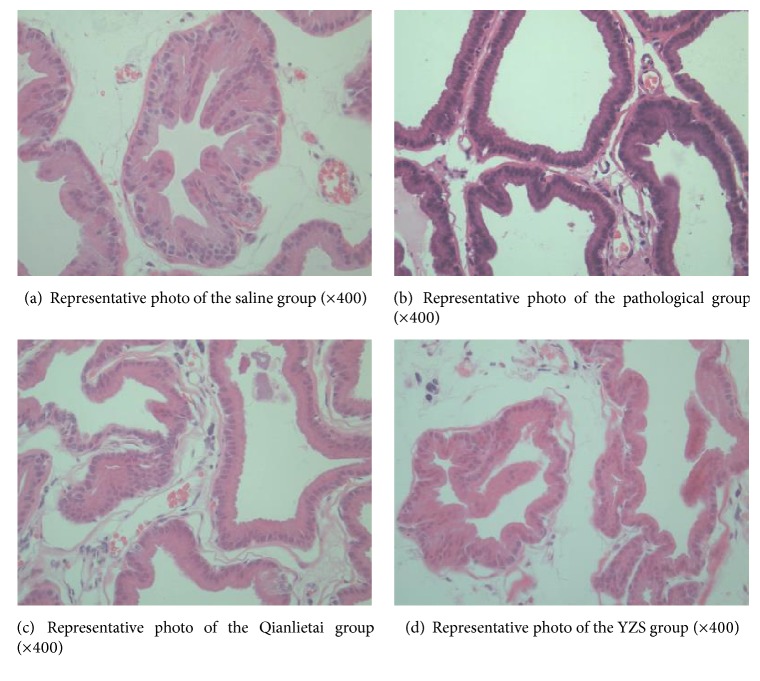
Representative histopathological photos following different treatments.

As shown in Figure 5, the volume of the prostate gland bubble was large in the saline group; the glandular epithelium had a flower-like appearance and protruded to the gland lumen; epithelial cells were single-ordered; the nuclei were small and round; arrays were present at the base of the epithelium; the cytoplasm was abundantly stained with red dye; a small amount of smooth muscle and fibre connective tissue could be observed in the mesenchyme; and no inflammatory cell infiltration was observed (Figure 5(a)). In the pathological model group, the prostate gland cavity was round or oval; the secretions were significantly reduced in the gland lumen; the glandular epithelium was atrophied; the nuclei were round and closely arranged; the cytoplasm was basophilic; the capillaries proliferated; dilation, congestion, and scattering were observed in the lymphocyte infiltration; and fibrous tissue hyperplasia was observed (Figure 5(b)). Most of the prostate acini epithelium returned to normal in the Qianlietai group and the YZS group, and most lumens were larger than those in the pathological model group. The glandular epithelium cells were arranged in high columns, and secretions could be observed in the gland lumen, which was most significant in the YZS group. Capillary proliferation dilation and congestion could always be observed in the Qianlietai group. No inflammatory cell infiltration was observed in the two groups. Proliferation of the fibre structure was clearly reduced (Figures 5(c) and 5(d)).

### 3.5. General Histopathology Injury Score

**Figure 6:**
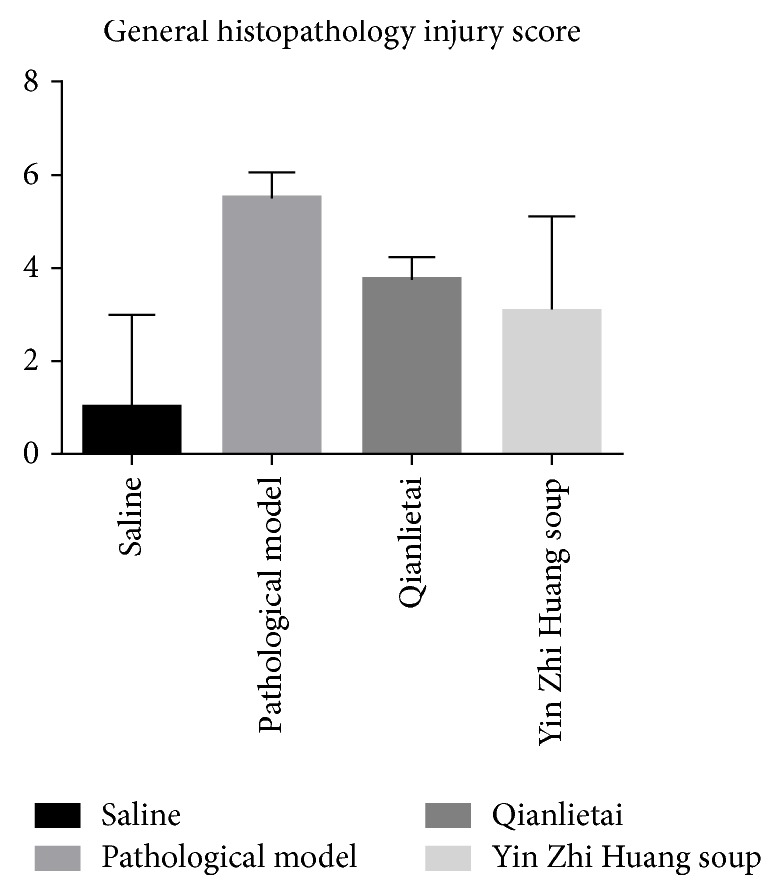


Significant differences were observed among the YZS group, the Qianlietai group, the saline group, and the pathological model group using the EAP model pathological grading standard score (*P* < 0.05). Compared with the Qianlietai group, the YZS group and pathological model group exhibited decreases in the measured values, and the greatest decreases were observed in the YZS group (see Figure 6).

The scores of the 4 groups significantly differed, and significant differences were observed between all pairs of groups (*P* < 0.05), as shown in Figure 6.

## 4. Discussion

Several recent clinical studies have demonstrated that YZS has good anti-inflammatory effects against CP/CPPS [13, 24]. However, the effects of YZS on reducing TNF-*α* content in rat serum and iNOS concentration in prostate tissues have not yet been reported. The results of the present study demonstrated that treatment with YZS decreased the expression of proinflammatory cytokines, such as TNF-*α*, and chemokines, such as iNOS, in a rat model of EAP. Our major findings revealed that TNF-*α* levels in the blood serum increased as a result of the DPT vaccine delivered via an intraperitoneal injection and the administration of purified prostate protein and FCA (1 : 1 ratio of the suspension) by multipoint intradermal injections. These treatments should increase the expression of iNOS in the prostate and induce injury in the rats. The prostate wet weight and the ratio of the prostate wet weight to body weight in the pathological model group were higher than those in the other groups, and the pathological model rats exhibited fidgeting behaviour, increased fighting behaviour, reduced eating and drinking, small amounts of yellow urine, and reduced stool output. Pathological examination revealed morphological damage to the prostate tissue and infiltration of inflammatory cells in the pathological model group, but there were no obvious changes in the saline group. After the YZS treatment, most of the prostate acini epithelium returned to normal and no inflammatory cell infiltration was observed in the YZS group. Proliferation of the fibre structure was clearly reduced under the light microscope. YZS reduced TNF-*α* levels in the blood serum and inhibited iNOS expression in the prostate tissues of the EAP rats. TNF-*α* levels and iNOS expression were also higher in the pathological model group than in the Qianlietai group. YZS improved the immune function and reduced the wet weight and ratio of prostate wet weight to body weight. Furthermore, the changes in the YZS group were significantly better than those in the Qianlietai group.

Many studies have shown that people are concerned with and pay more attention to the effects of abnormal immune responses during disease development, such as CP/CPPS. Internal and external environmental changes can lead to partial prostate-specific antigen (PSA) exposure, which then stimulates CD^+^_4_T in blood and tissues. Active immune cells directly or indirectly infiltrate the prostate tissue [25]. Proinflammatory cytokines, such as TNF-*α*, and chemokines, such as iNOS, interleukin-6 (IL-6), and IL-8, produce varying degrees of improvement [26, 27].

TNF-*α* is secreted by macrophages and lymphocyte cytokines. TNF-*α* can activate neutrophils and mononuclear macrophages to promote the release of proinflammatory factors, thereby promoting inflammatory responses [28]. Li et al. [29] showed that TNF-*α* was significantly increased in the serum and prostate tissue of autoimmune prostate rats. Pontari and Ruggieri [30] reported high levels of TNF-*α* in prostatic fluid from patients with CP/CPPS, which is indicative of persistent inflammation in these patients. The NO concentration and iNOS expression in CP/CPPS patients are significantly higher than those in normal controls. NO is synthesized from L-arginine through NOS. Under normal circumstances, endothelial NO has an inhibitory effect on inflammatory responses, but in pathological states, iNOS synthesis produces a large amount of NO, which has a cytotoxic effect and increases the inflammatory response [31]. iNOS is an important inflammatory gene expression product that is mainly distributed in the cytoplasm of prostate cells. iNOS expression increases, synthesizes a large amount of NO, and participates in inflammatory responses. Furthermore, NO can react with peroxides and peroxodisulfates, which promotes cytotoxic and DNA damage [32]. Therefore, TNF-*α* and iNOS play important roles in immune regulation and the incidence of CP/CPPS.

CP/CPPS patients can present with a wide range of clinical manifestations. The four main symptom domains are lower urinary tract symptoms (LUTS, voiding or storage symptoms), urogenital pain, erectile dysfunction (ED), and psychological issues [33]. A recent multicentre study determined that the prevalence of ED in patients with CP/CPPS was 45.4%; LUTS and ED may have common pathological bases, including the nitric oxide synthase/nitric oxide theory and autonomic nervous system hyperfunction and metabolic syndrome hypothesis and the pelvic arteriosclerosis theory [19]. An increasing body of clinical evidence has indicated that the inflammatory process is likely associated with LUTS in patients with CP/CPPS, and LUTS and erectile function are closely linked. In terms of histological prostatitis, an association has been reported between decreased erectile function and histological inflammation of the prostate tissue [20, 34]. Kumsar et al. revealed that inflammation and benign prostatic hyperplasia may also affect erectile function via similar mechanisms [21]. The pathogenesis of endothelial dysfunction includes decreased vascular NO bioavailability, impaired vasodilatation, enhanced inflammation, and oxidative stress with the proliferation of smooth muscle cells [22].

YZS is a classic TCM in China and was recorded in a clinical TCM book titled Shang Han Lun. YZS comprises seven Chinese herbs: herbal* Artemisia scoparia*,* Rheum palmatum* L., Fructus Gardeniae,* Artemisia anomala* S. Moore, Flos Chrysanthemi Indici, Semen Litchi, and* Taraxacum mongolicum* Hand.-Mazz, and it is a basic prescription for the treatment of clear damp-heat. YZS is a sophisticated compound that contains 7 Chinese medicinal herbs with a large content of monomers, including phenolic and flavonoid compounds and emodin, which have been shown to have anti-inflammatory effects in CP/CPPS. The primary active ingredients in YZS are listed in Table 1. Whether the anti-inflammatory effects of YZS are achieved by single or multiple components in YZS remains unclear. Jang et al. reported that herbal* Artemisia scoparia* exerts high levels of anti-inflammatory activity, and the extract of* Artemisia capillaris* suppresses NO production via the downregulation of iNOS transcription [23]. Pharmacological studies have revealed that* Rheum palmatum* L. has numerous pharmacological activities, such as anti-inflammatory, antimicrobial, antifungal, antivirus, and immunoenhancing activities [35]. Emodin (1,3,8-trihydroxy-6-methyl anthraquinone) is an anthraquinone derivative of the Chinese herb* Rheum palmatum* L. Emodin can decrease the increased levels of the proinflammatory cytokine TNF-*α* in rat plasma caused by lipopolysaccharide challenge [36]. Fructus Gardeniae is used for the treatment of inflammation, jaundice, headache, oedema, fever, and hepatic disorders. Fructus Gardeniae protects against oxidative damage and cytotoxic effects and has anti-inflammatory activity [37]. Xi et al. [38] demonstrated that* Artemisia anomala* S. Moore can inhibit iNOS-induced NO production and that an ethyl acetate extraction of* Artemisia anomala* S. Moore exhibited a strong inhibitory effect on LPS/IFN-*γ*-induced NO production and proinflammatory cytokine expression. Wu et al. [39] reported that Flos Chrysanthemi Indici decreases MDA levels by increasing the activities of anti-oxidant enzymes (SOD, GPx, and GRd), attenuating the production of NF-kB, TNF-*α*, IL-1*β*, IL-6, PGE2, and NO and suppressing the activities of iNOS and COX-2. Ahn et al. [40] concluded that a phenolic product called Oligonol derived from Semen Litchi had protective activity against oxidative stress-induced inflammation. Oligonol inhibited NO and ROS formation under cellular oxidative stress in C6 glial cells. Treatment with Oligonol also resulted in the downregulation of cyclooxygenase-2 (COX-2) and iNOS.

In our study, the mechanism of YZS was not fully clarified. We demonstrated that YZS reduces TNF-*α* content in blood serum and iNOS concentrations in prostate tissue in rats. Thus, YZS may have potential therapeutic effects on LUTS and ED. However, the identification and clarification of the effective components in YZS require further experiments. Because purified prostatic proteins were used to induce EAP in the Wistar rats in a manner that is histologically similar to CP/CPPS in humans, YZS is expected to be an effective therapy for the prevention and treatment of CP/CPPS in human patients.

## 5. Conclusions

Our study suggests that the classic TCM formula YZS had anti-inflammatory effects in an EAP Wistar rat model. YZS treatment reduced inflammatory changes in the prostate tissue. It also significantly suppressed proinflammatory cytokines, such as TNF-*α*, and chemokines, such as iNOS, in the rat model of EAP. Therefore, YZS may have potential therapeutic effects on LUTS and ED, and this classic TCM formula may be effective for the clinical treatment of CP/CPPS in humans. Furthermore, because YZS consists of multiple components, further studies should focus on identifying the primary effective biomonomer.

## Figures and Tables

**Table 1:** Primary active ingredients and functions of Chinese herbs.

Chinese herbs	Active ingredients	Functions	Ref.
*Artemisia scoparia*	Phenolic and flavonoid compounds	Suppresses NO production via the downregulation of iNOS transcription	[19]
*Rheum palmatum* L.	Emodin	Decreases the proinflammatory cytokine TNF-*α* in rat plasma	[20]
Fructus Gardeniae	Phenolic and flavonoid compounds	Antioxidative damage effects, cytotoxic effects, and anti-inflammatory activity	[21]
Flos Chrysanthemi Indici	Flavonoids, terpenoids, and phenolic compounds	Suppresses the activities of iNOS and COX-2	[22]
Semen Litchi	Oligonol	Inhibits NO and ROS formation	[23]

## Abbreviations

YZS: Yin Zhi Huang soup
EAP: Experimental autoimmune prostatitis
TNF-*α*: Tumor necrosis factor-alpha
iNOS: Inducible nitric oxide synthase
CP/CPPS: Chronic prostatitis/chronic pelvic pain syndrome
NO: Nitric oxide
TCM: Traditional Chinese Medicine
DPT: Diphtheria, acellular pertussis, and tetanus combined
FCA: Freund's complete adjuvant
PBS: Phosphate-buffered salt
ED: Erectile dysfunction
LUTS: Lower urinary tract symptoms.

## Supplementary Materials

Supplementary Materials Five original photos in Figure  5 and the description of their applications in the study.
