# Supplementary figures and images for: The Anti-Inflammatory Effects of a Yin Zhi Huang Soup in an Experimental Autoimmune Prostatitis Rat Model
Source: Evid Based Complement Alternat Med. 2017 Dec 21;2017:7312938. doi: 10.1155/2017/7312938 (PMC5752995; doi:10.1155/2017/7312938)

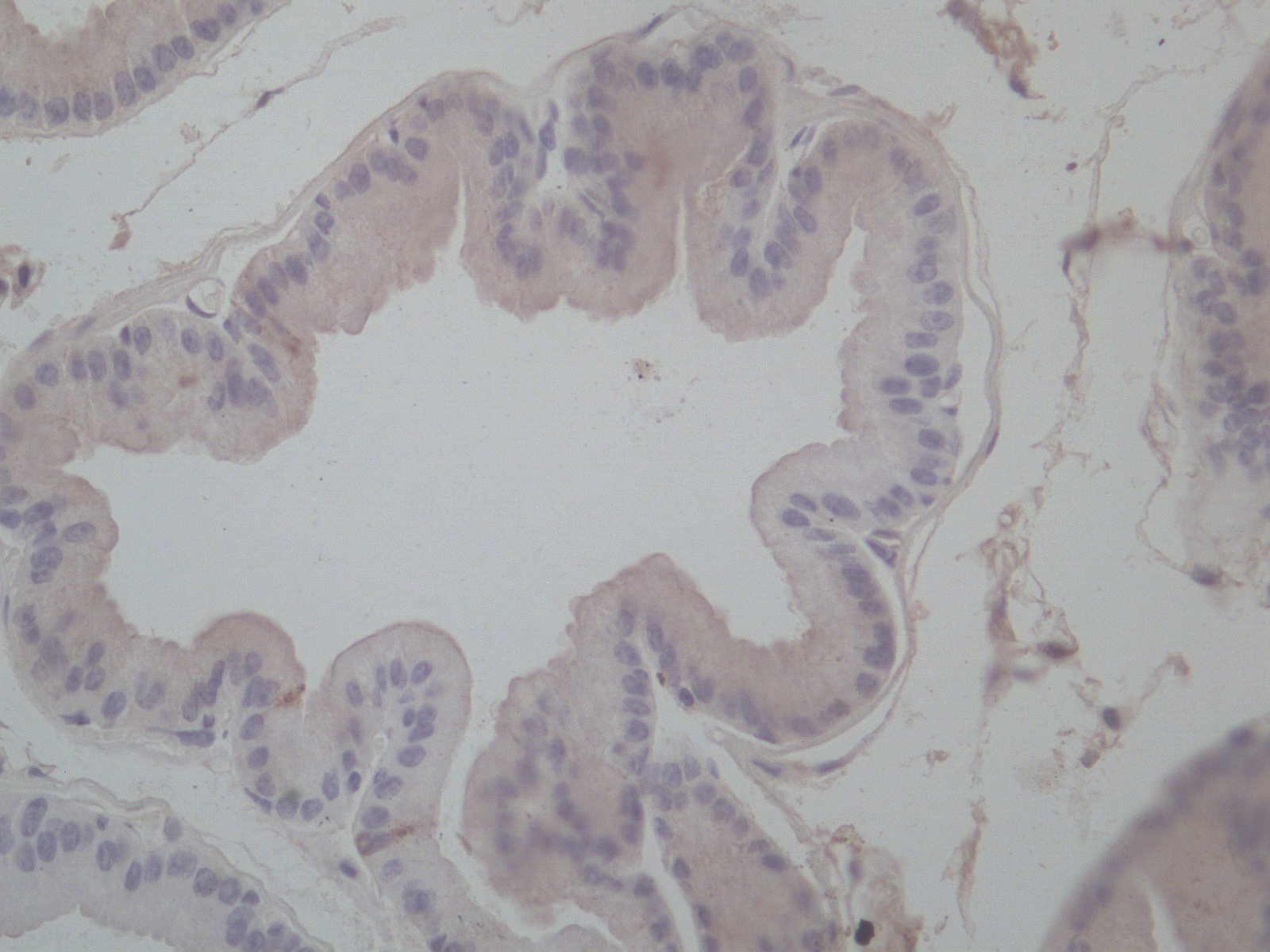

Supplement: Supplementary Materials — Five original photos in Figure 5 and the description of their applications in the study. [file 7312938.f1.zip › pathological model group/1.jpg]

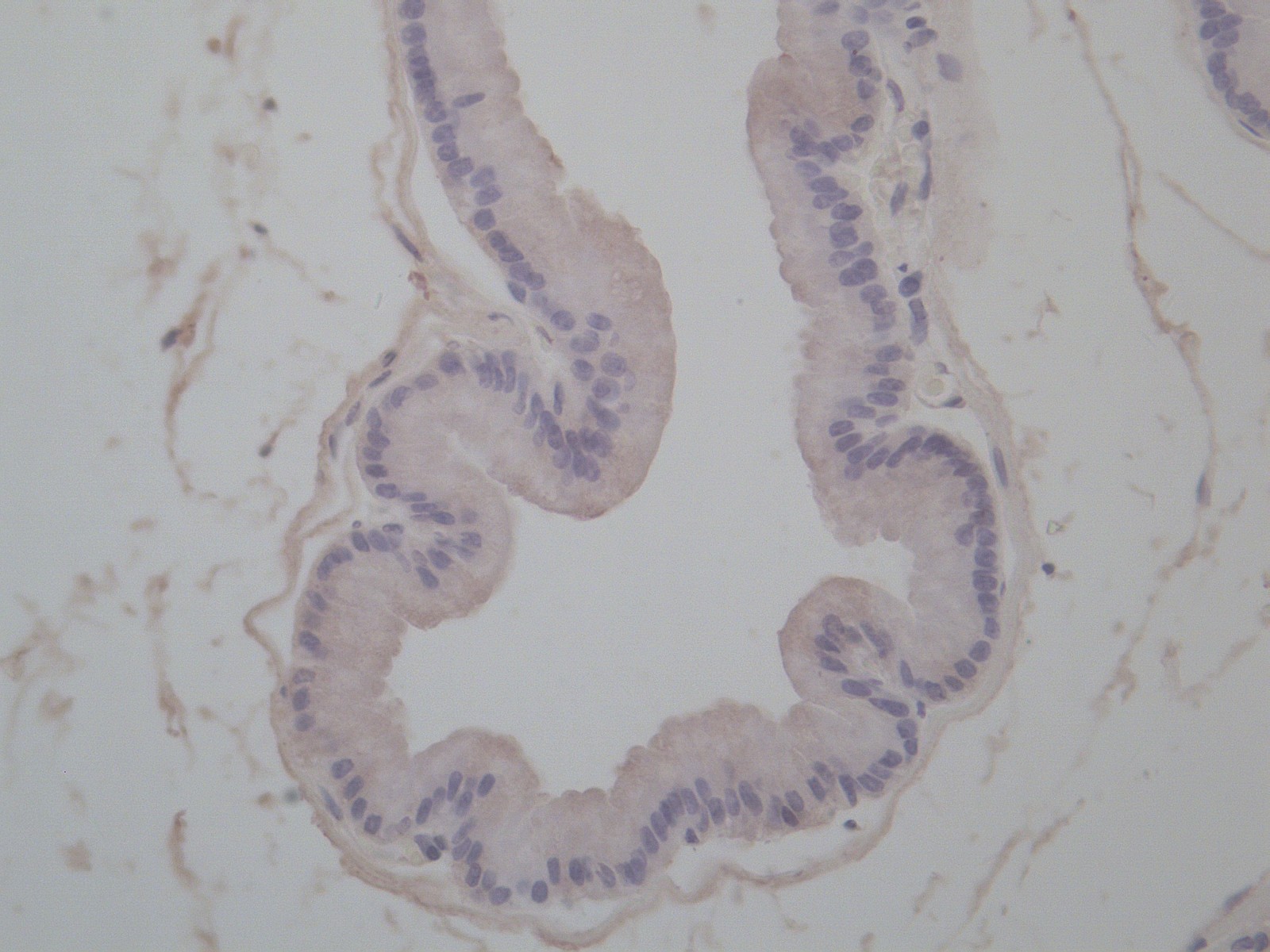

Supplement: Supplementary Materials — Five original photos in Figure 5 and the description of their applications in the study. [file 7312938.f1.zip › pathological model group/2.jpg]

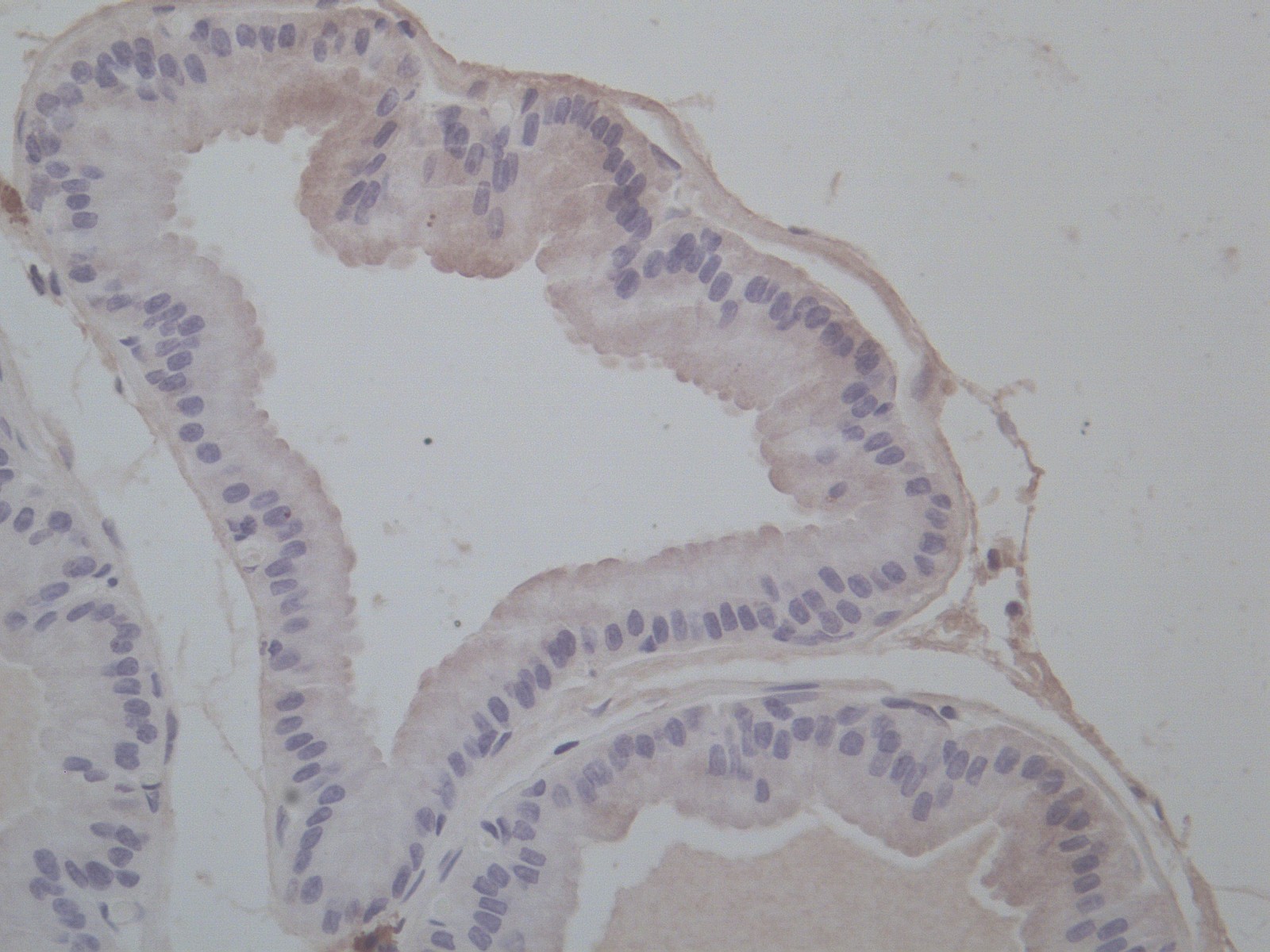

Supplement: Supplementary Materials — Five original photos in Figure 5 and the description of their applications in the study. [file 7312938.f1.zip › pathological model group/3.jpg]

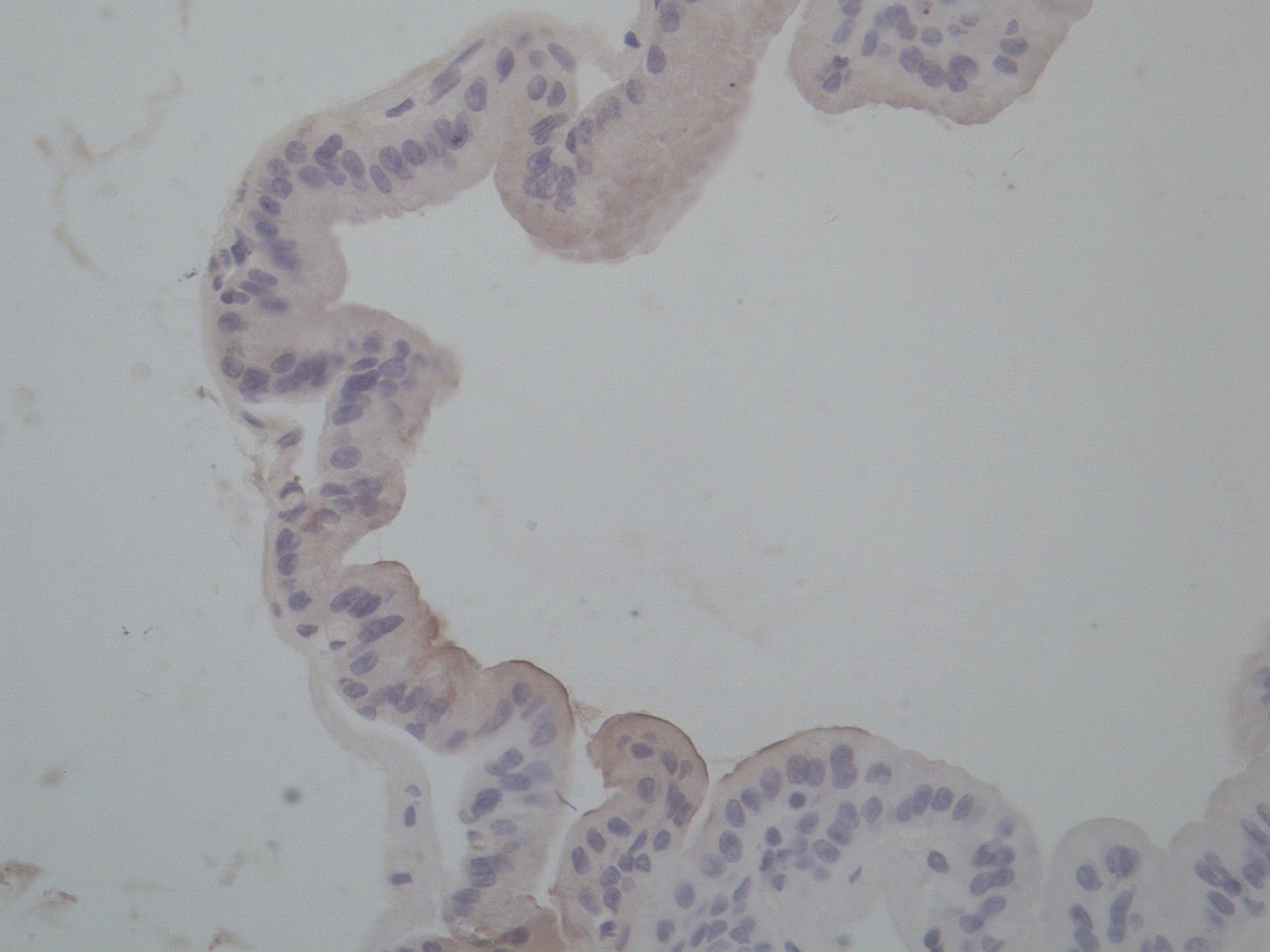

Supplement: Supplementary Materials — Five original photos in Figure 5 and the description of their applications in the study. [file 7312938.f1.zip › pathological model group/4.jpg]

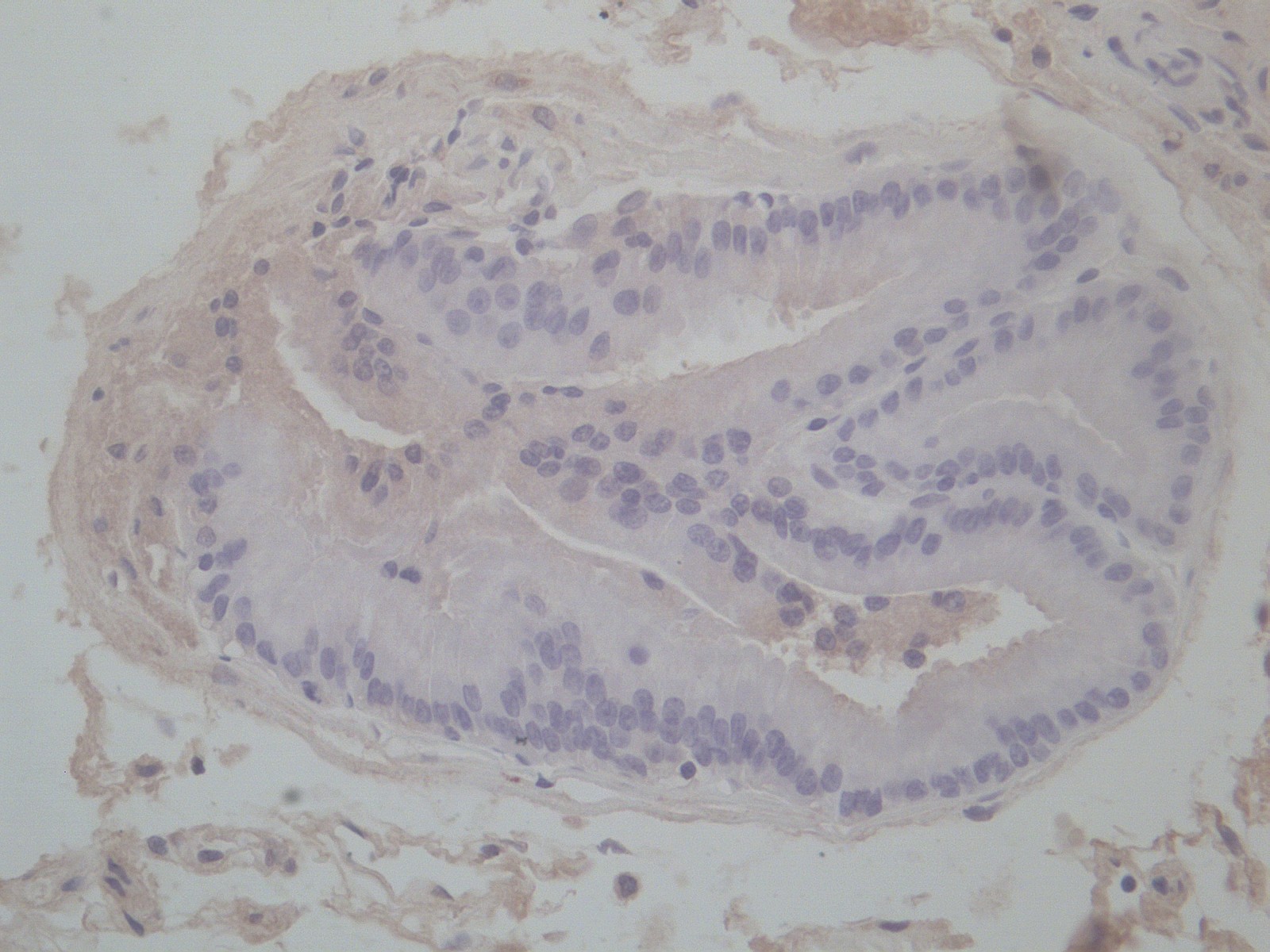

Supplement: Supplementary Materials — Five original photos in Figure 5 and the description of their applications in the study. [file 7312938.f1.zip › pathological model group/5.jpg]

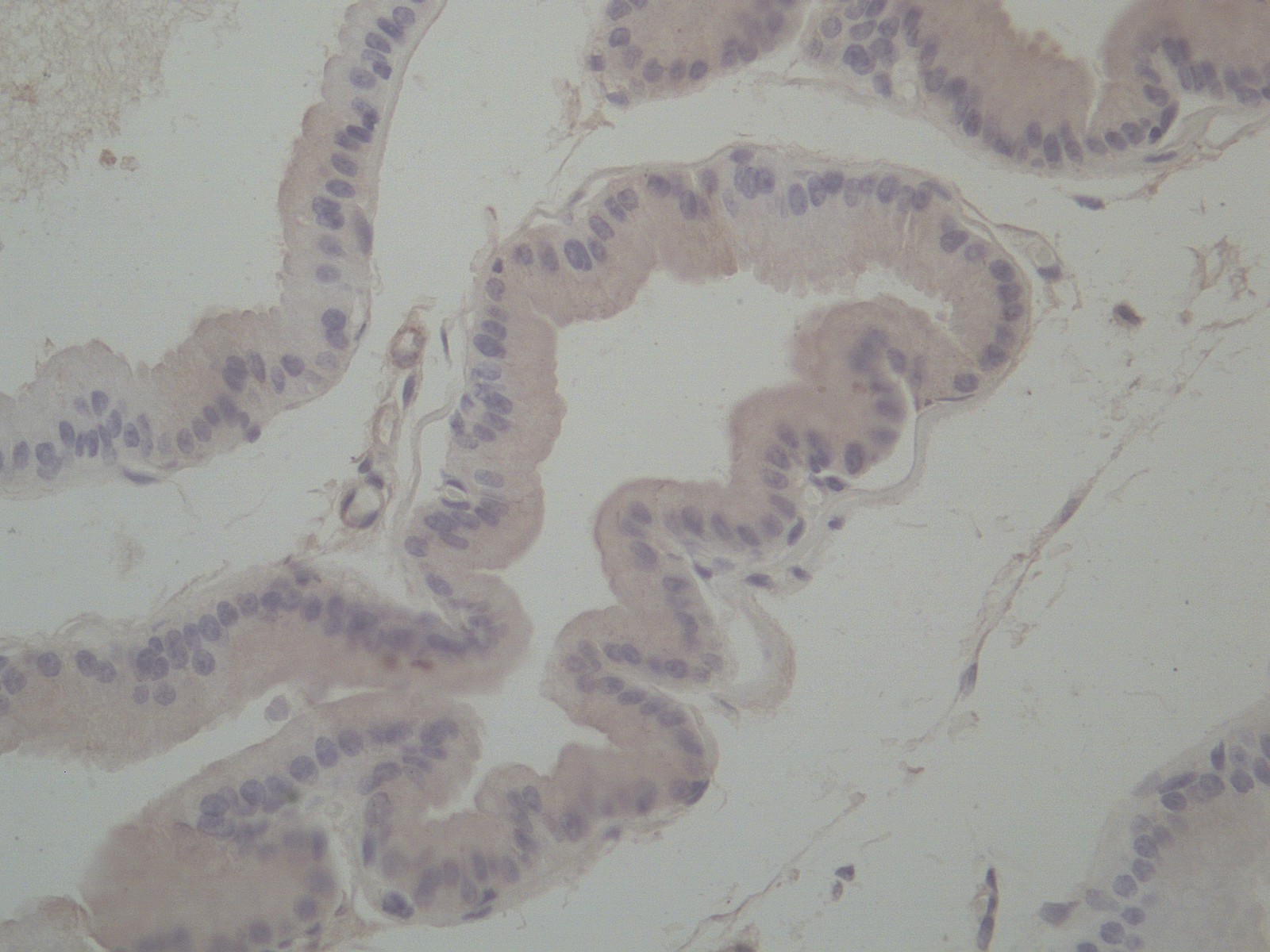

Supplement: Supplementary Materials — Five original photos in Figure 5 and the description of their applications in the study. [file 7312938.f1.zip › Qianlietai group/1.jpg]

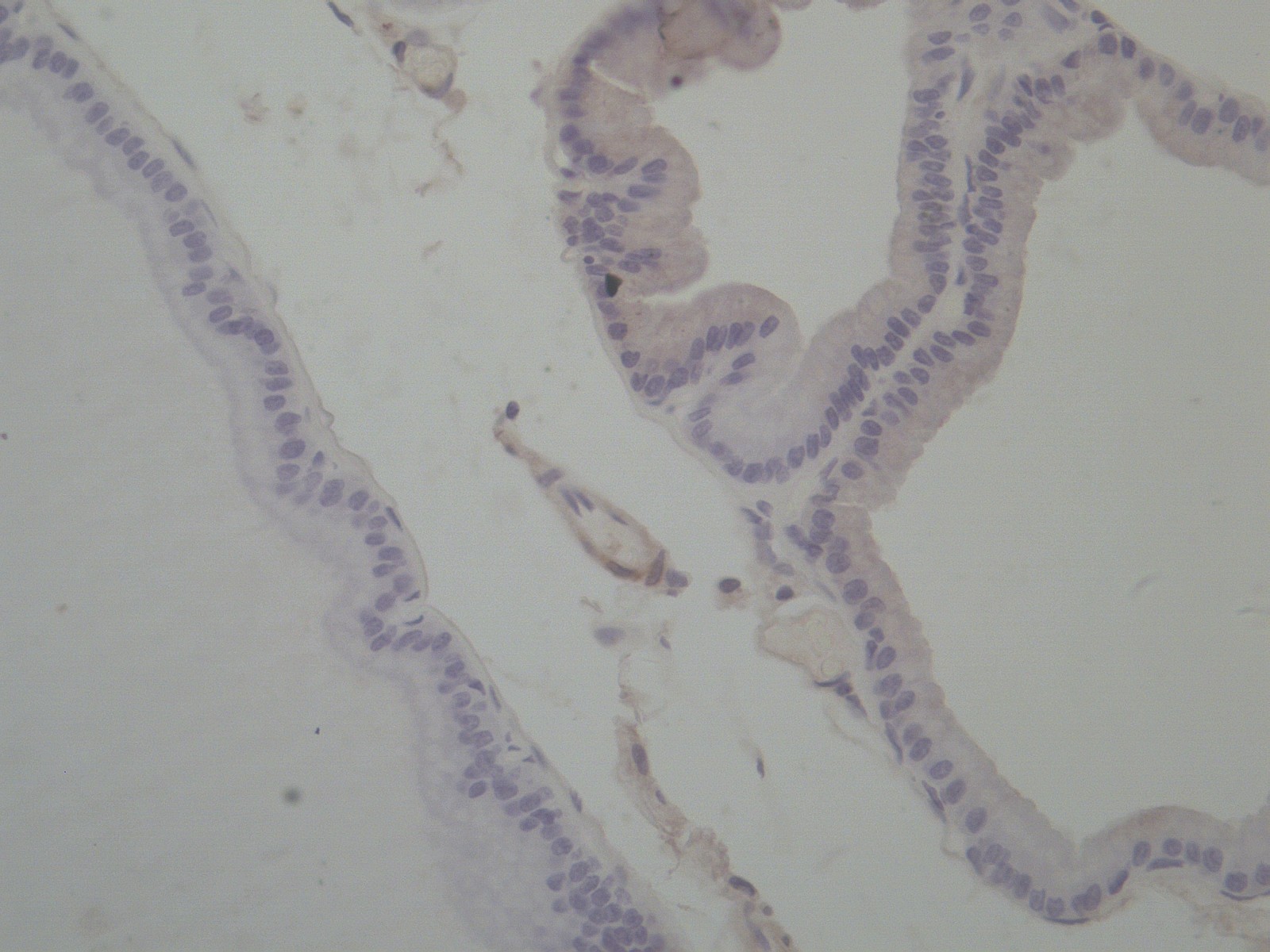

Supplement: Supplementary Materials — Five original photos in Figure 5 and the description of their applications in the study. [file 7312938.f1.zip › Qianlietai group/2.jpg]

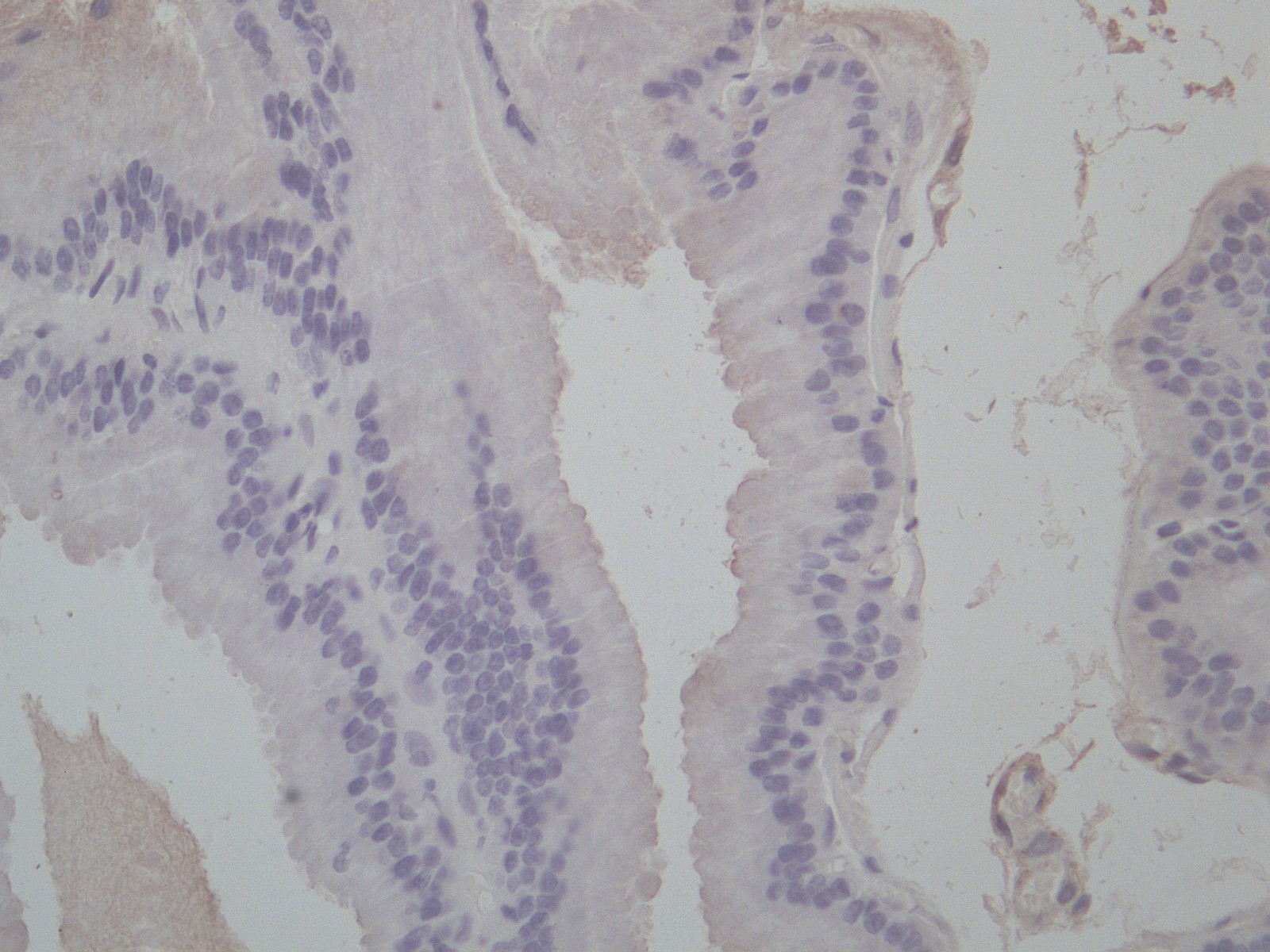

Supplement: Supplementary Materials — Five original photos in Figure 5 and the description of their applications in the study. [file 7312938.f1.zip › Qianlietai group/3.jpg]

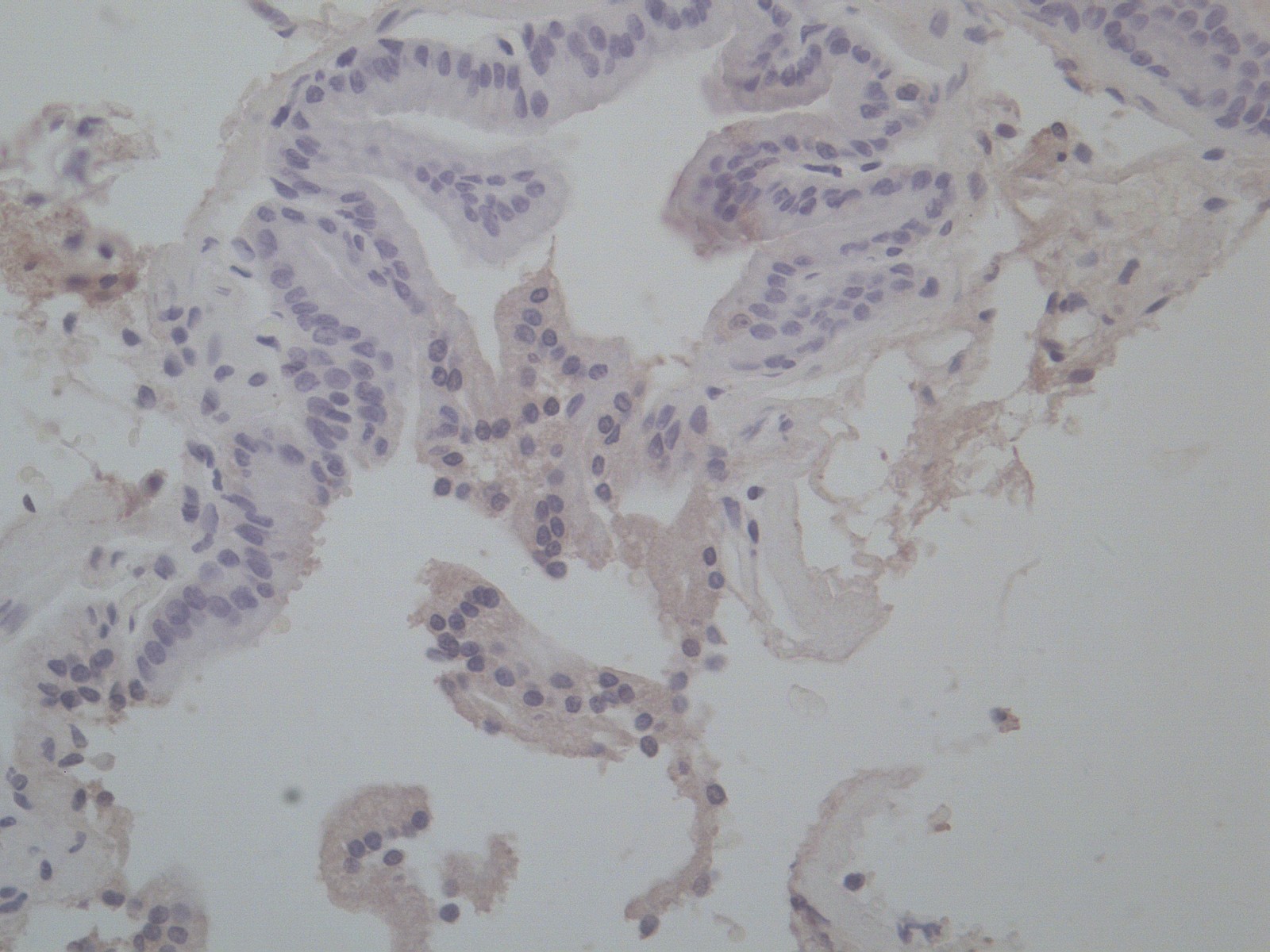

Supplement: Supplementary Materials — Five original photos in Figure 5 and the description of their applications in the study. [file 7312938.f1.zip › Qianlietai group/4.jpg]

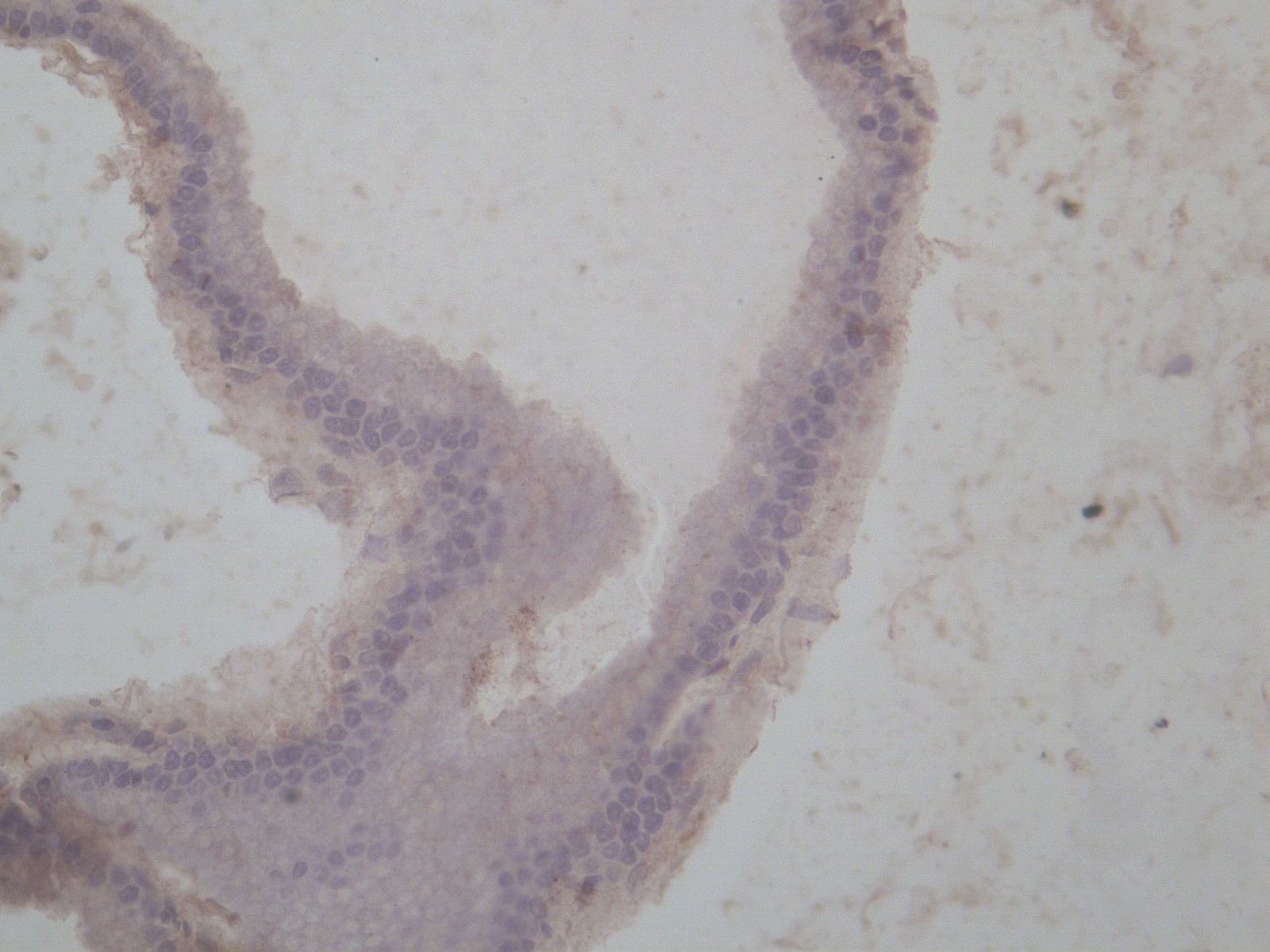

Supplement: Supplementary Materials — Five original photos in Figure 5 and the description of their applications in the study. [file 7312938.f1.zip › Qianlietai group/5.jpg]

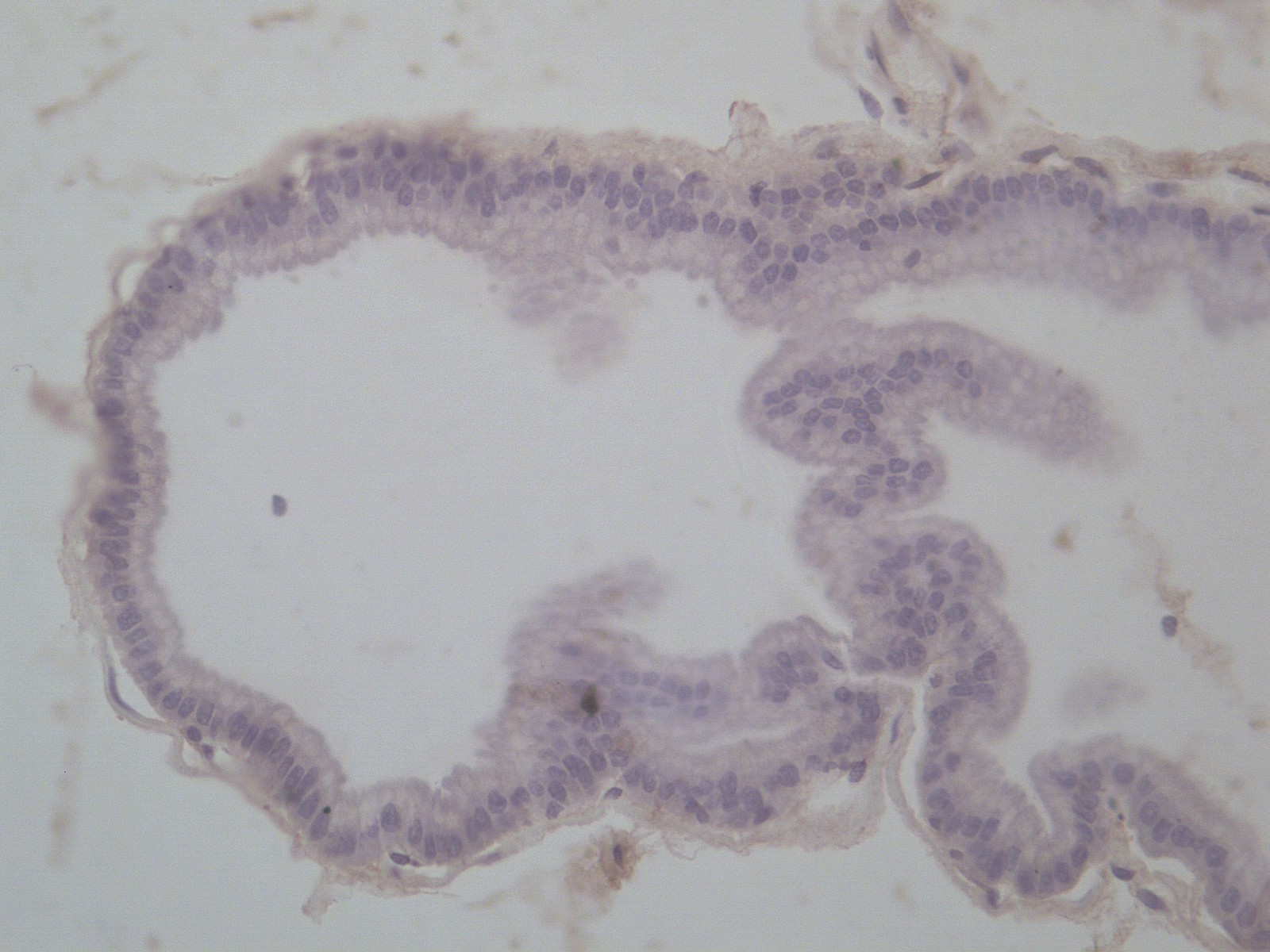

Supplement: Supplementary Materials — Five original photos in Figure 5 and the description of their applications in the study. [file 7312938.f1.zip › saline group/1.jpg]

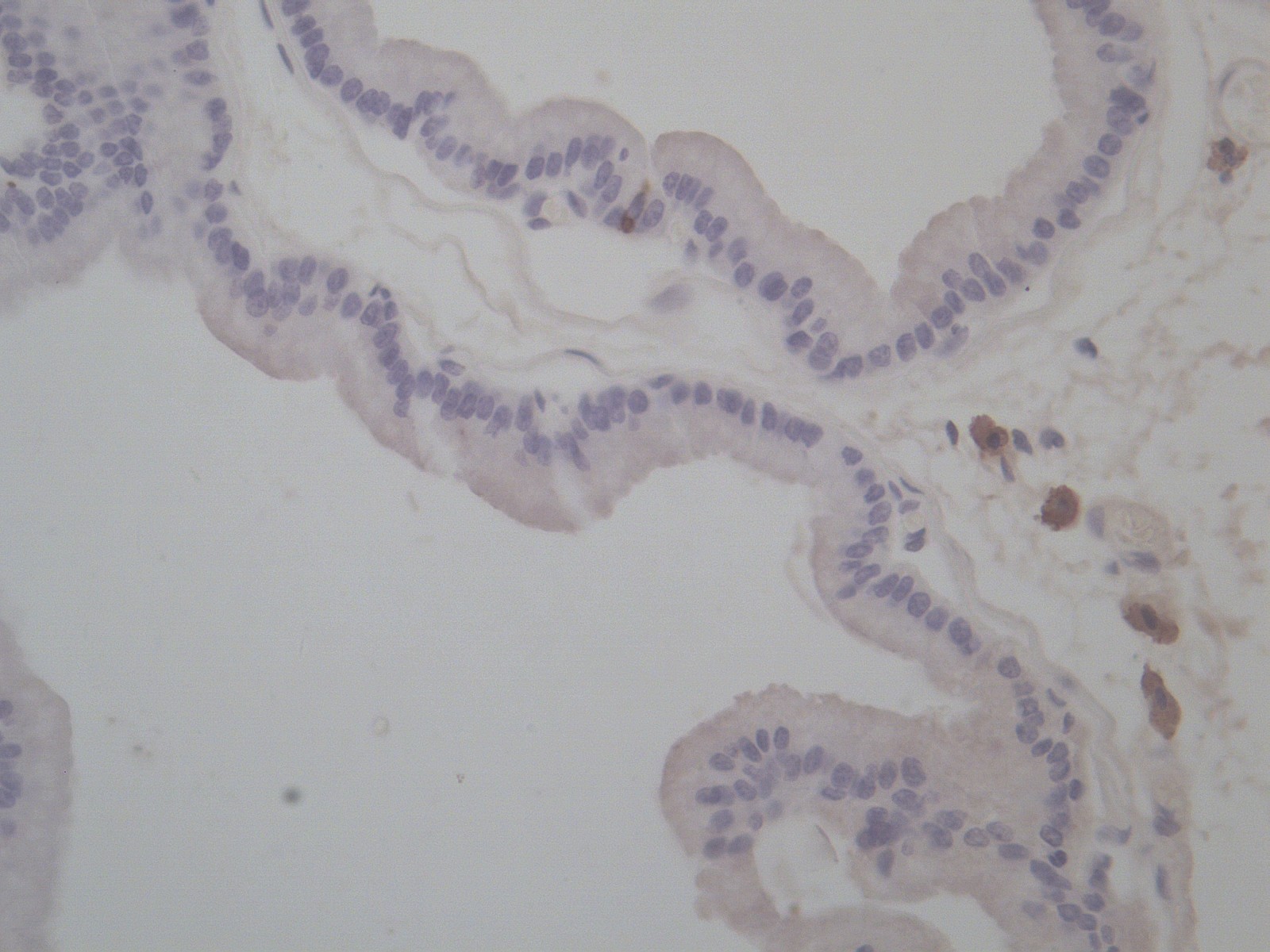

Supplement: Supplementary Materials — Five original photos in Figure 5 and the description of their applications in the study. [file 7312938.f1.zip › saline group/2.jpg]

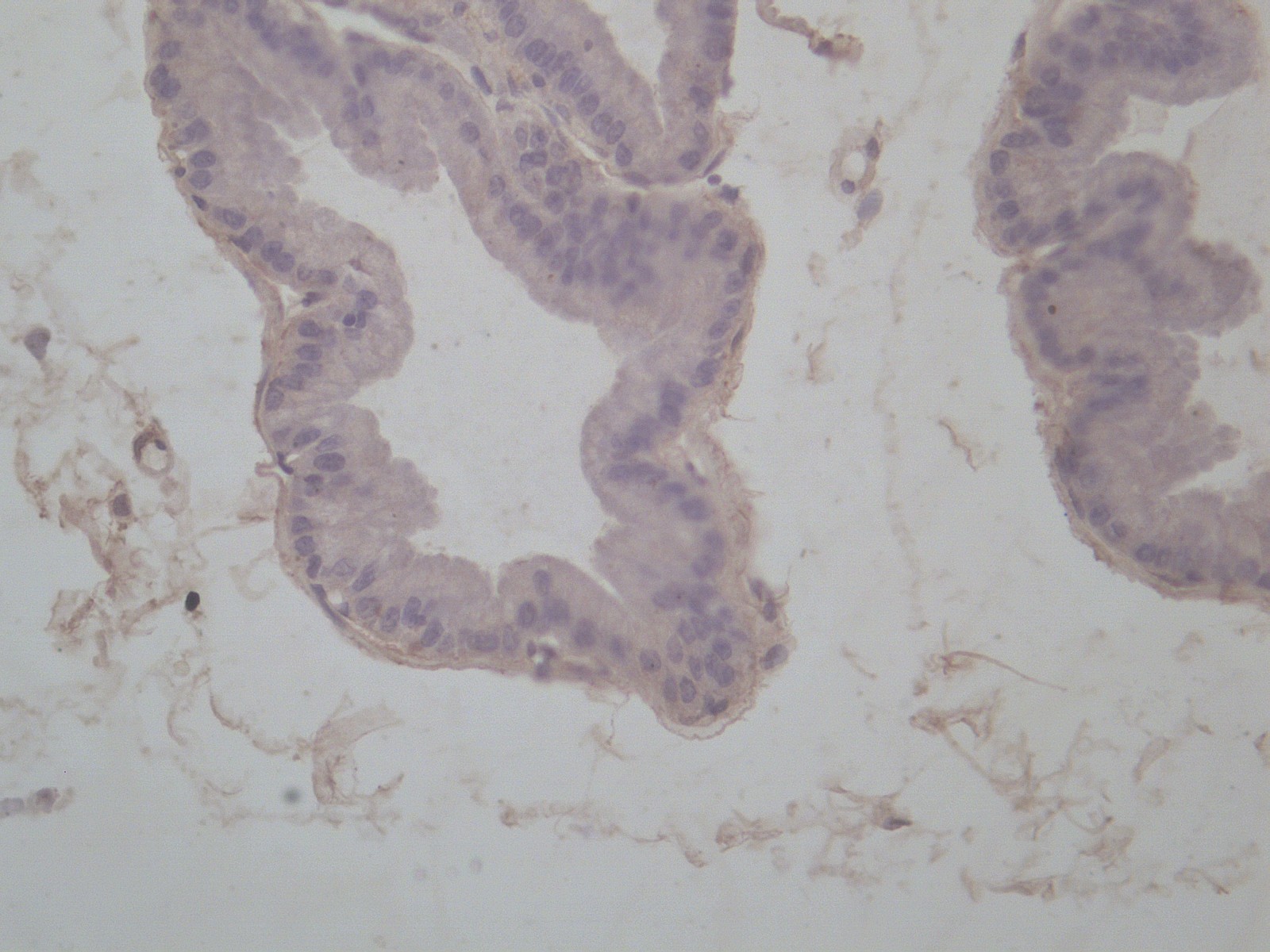

Supplement: Supplementary Materials — Five original photos in Figure 5 and the description of their applications in the study. [file 7312938.f1.zip › saline group/3.jpg]

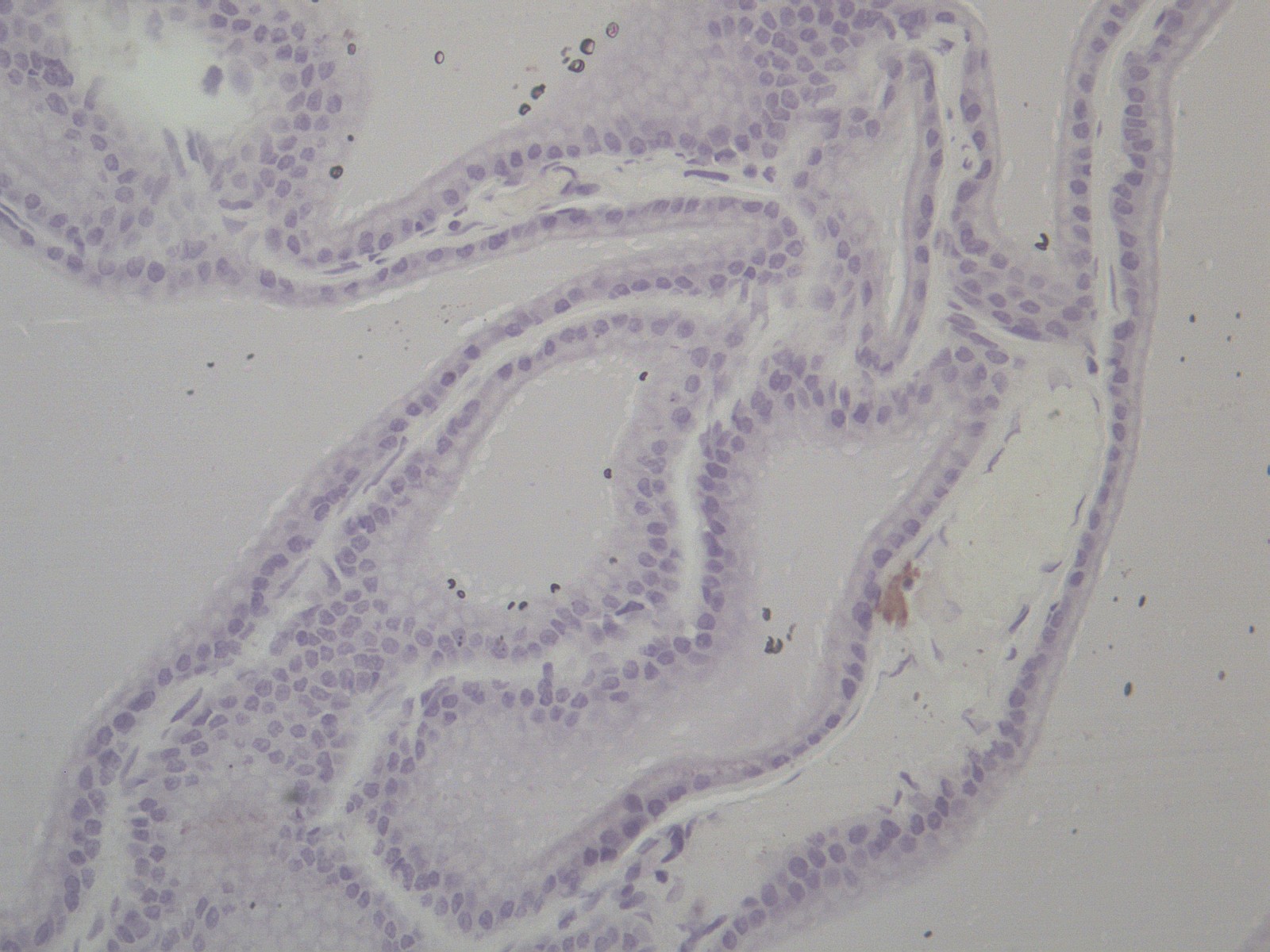

Supplement: Supplementary Materials — Five original photos in Figure 5 and the description of their applications in the study. [file 7312938.f1.zip › saline group/4.jpg]

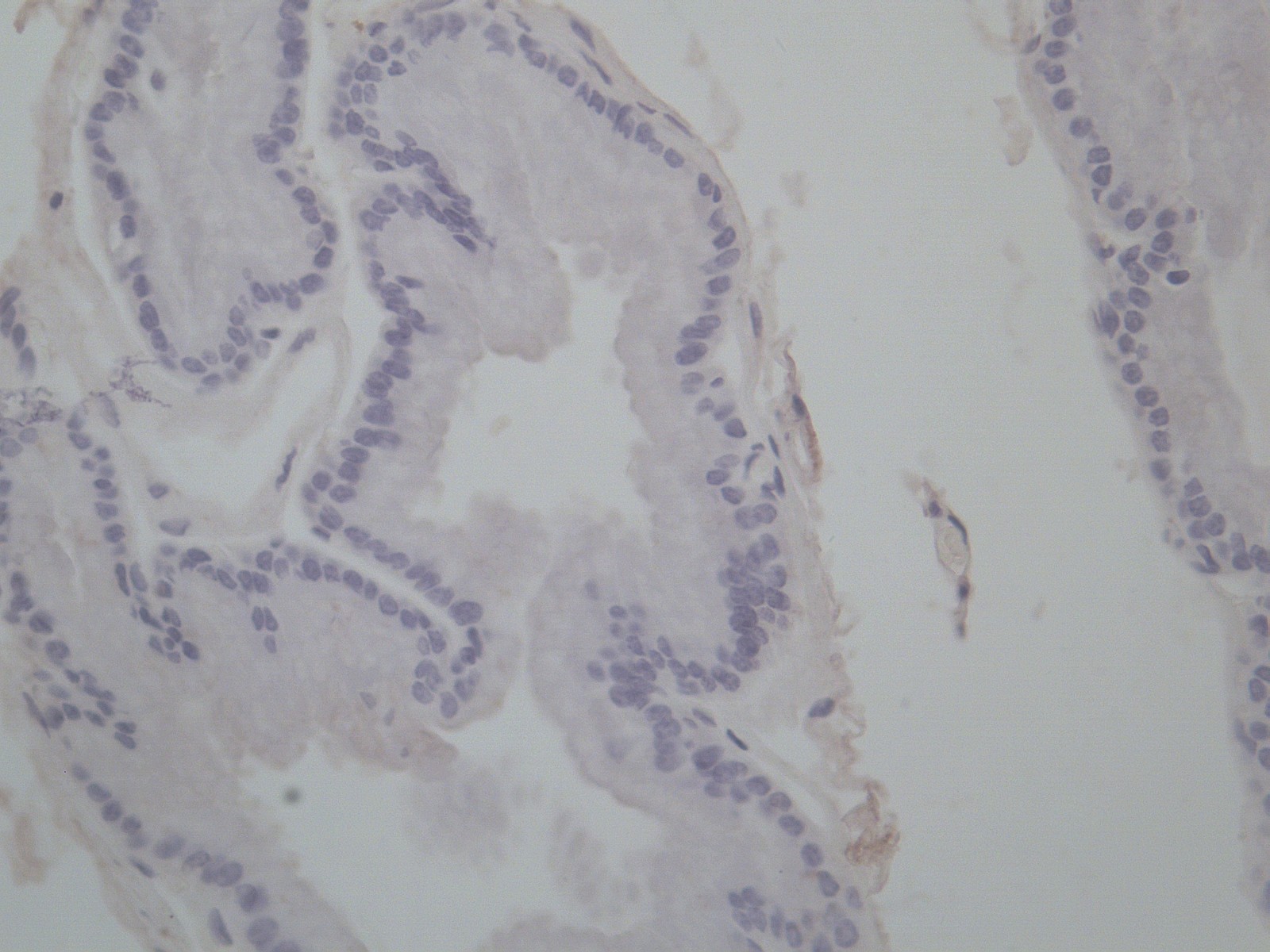

Supplement: Supplementary Materials — Five original photos in Figure 5 and the description of their applications in the study. [file 7312938.f1.zip › saline group/5.jpg]

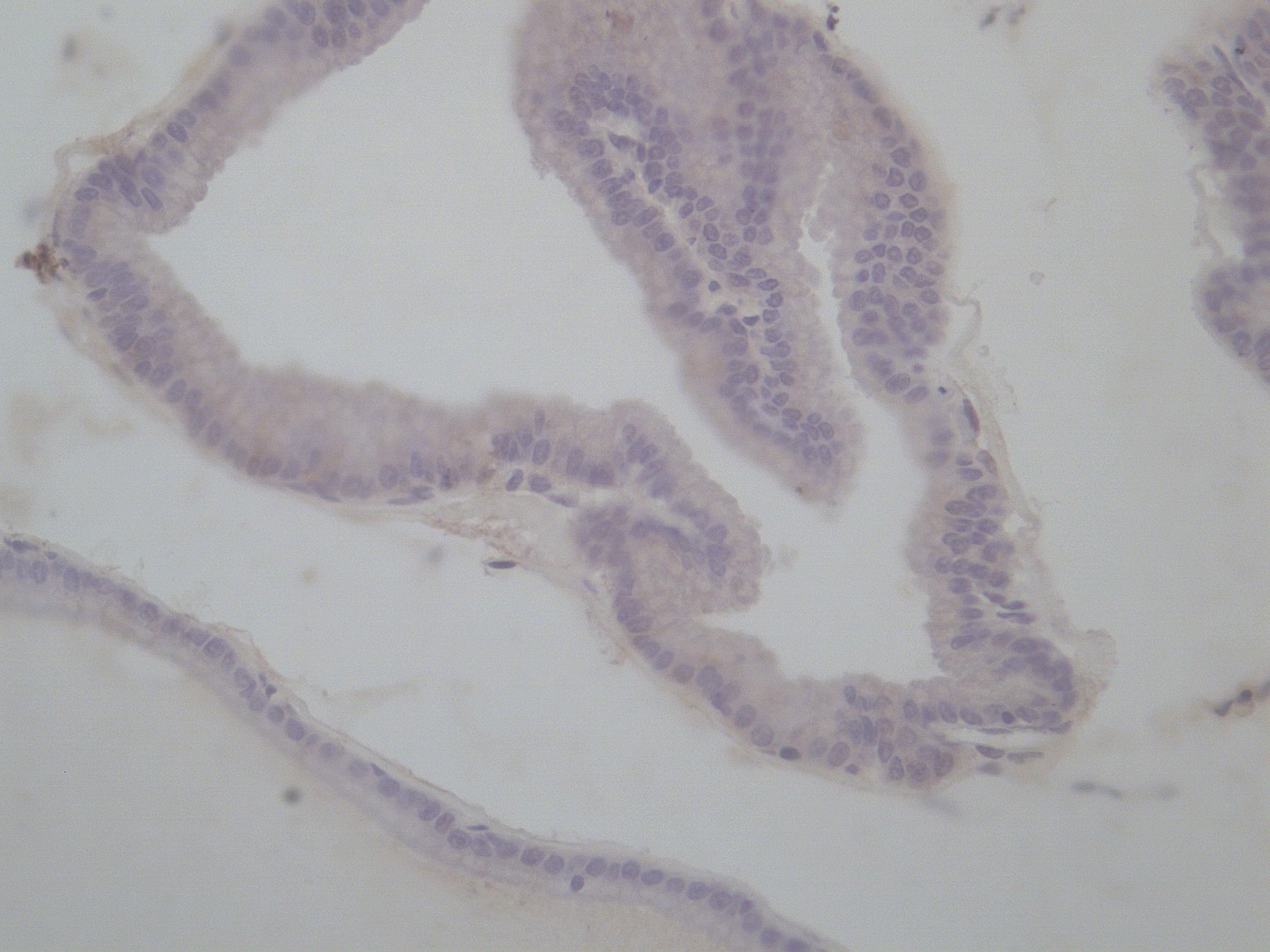

Supplement: Supplementary Materials — Five original photos in Figure 5 and the description of their applications in the study. [file 7312938.f1.zip › Yin Zhi Huang Soup group/1.jpg]

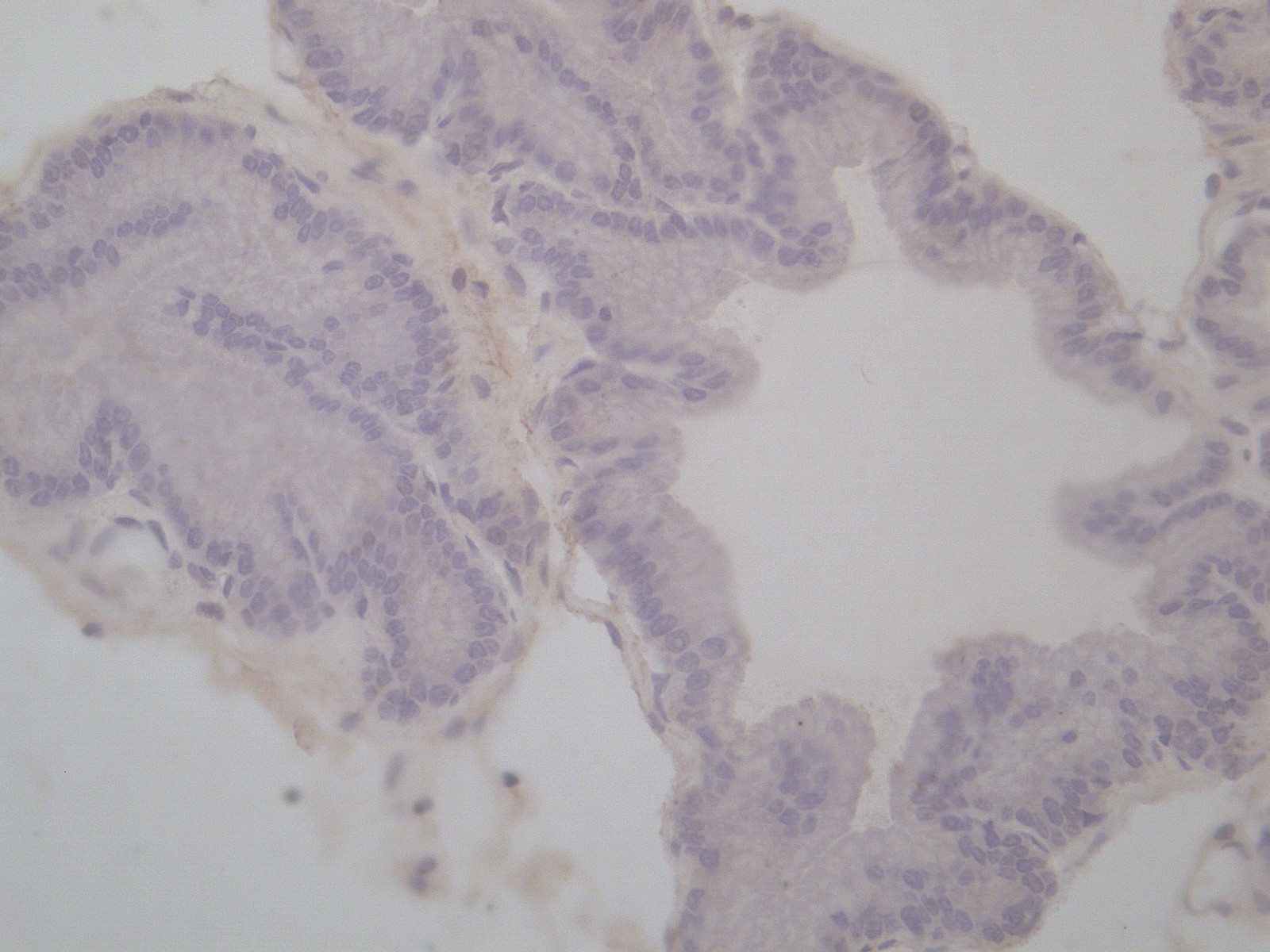

Supplement: Supplementary Materials — Five original photos in Figure 5 and the description of their applications in the study. [file 7312938.f1.zip › Yin Zhi Huang Soup group/2.jpg]

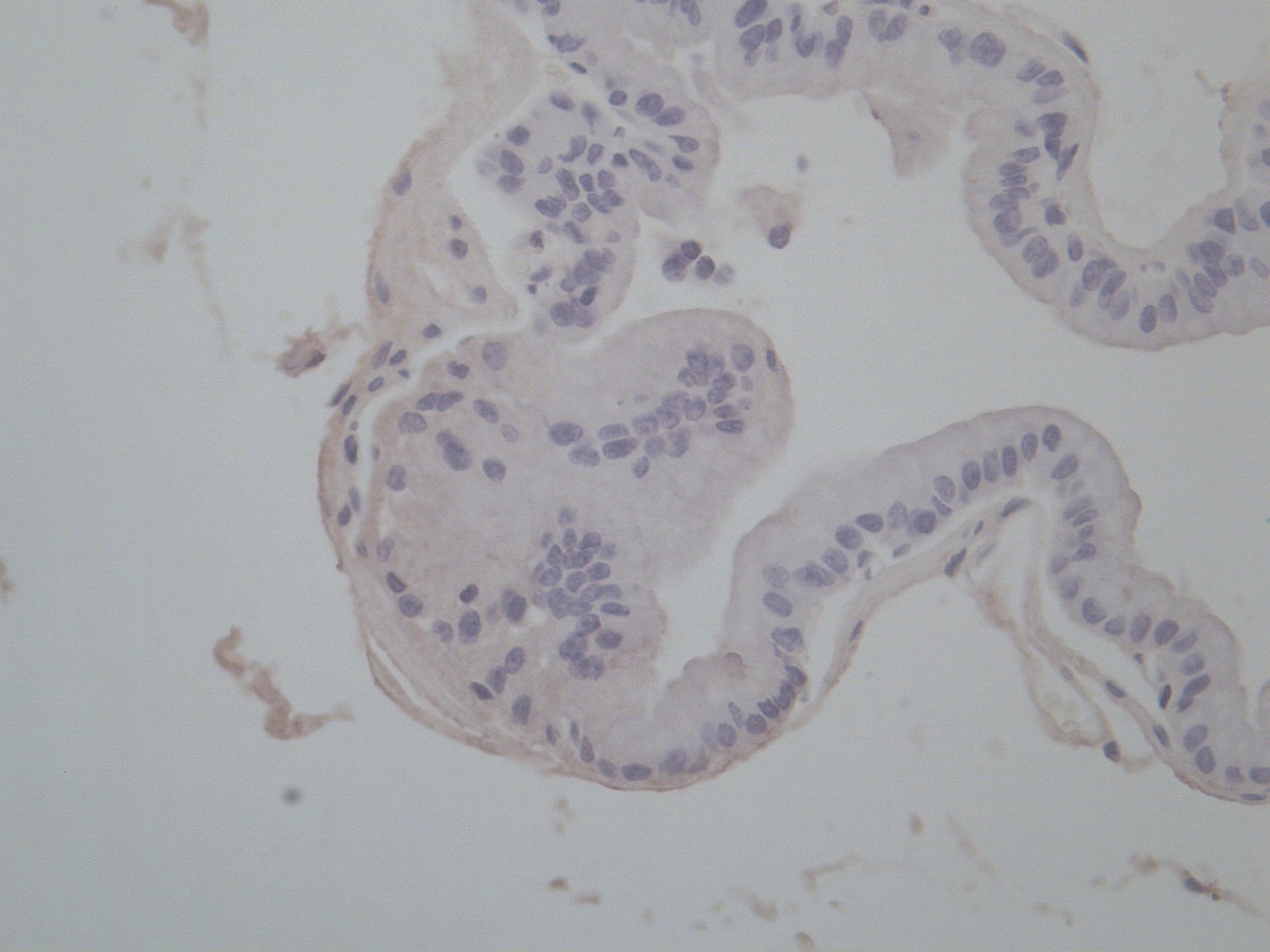

Supplement: Supplementary Materials — Five original photos in Figure 5 and the description of their applications in the study. [file 7312938.f1.zip › Yin Zhi Huang Soup group/3.jpg]

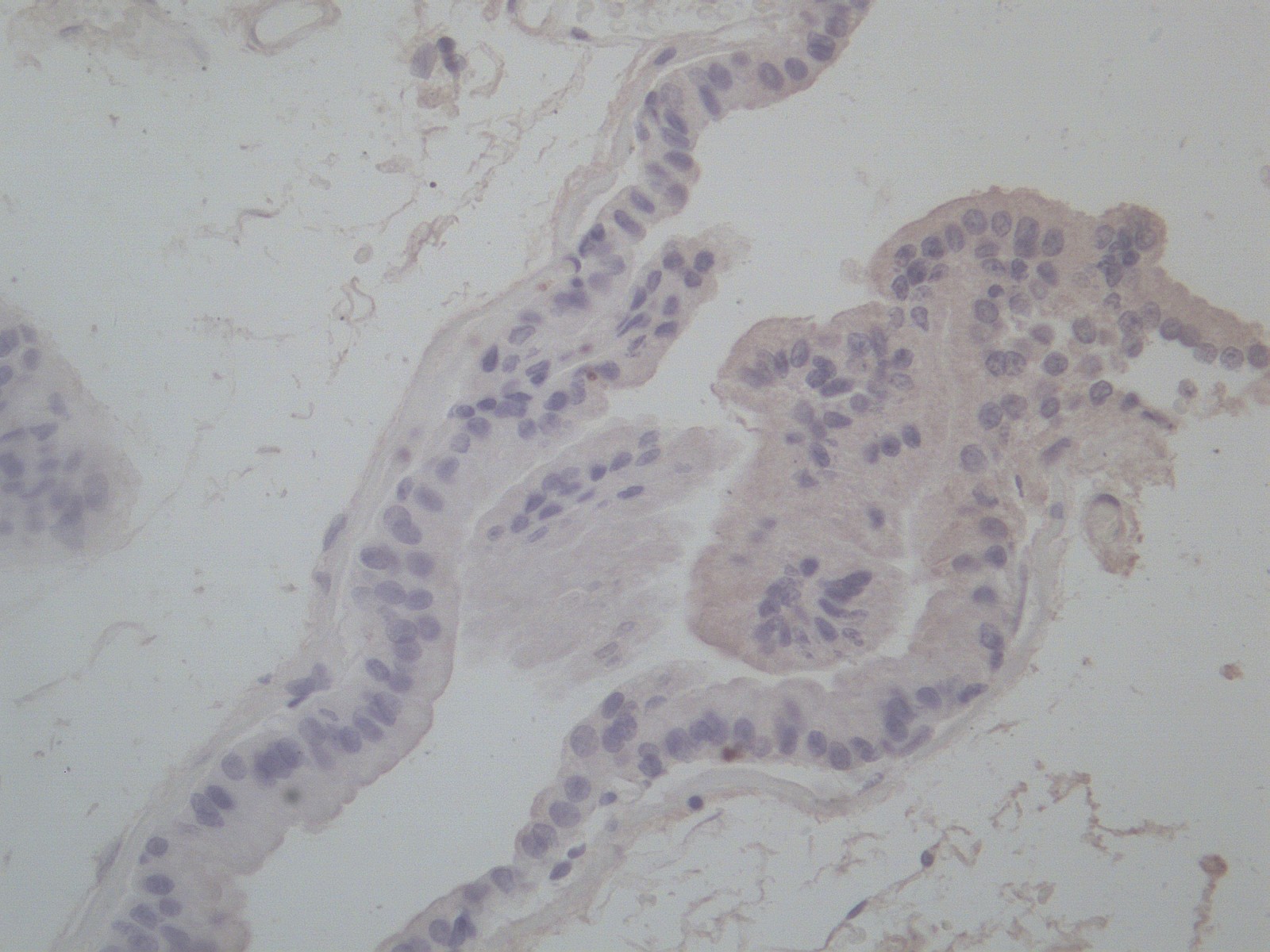

Supplement: Supplementary Materials — Five original photos in Figure 5 and the description of their applications in the study. [file 7312938.f1.zip › Yin Zhi Huang Soup group/4.jpg]

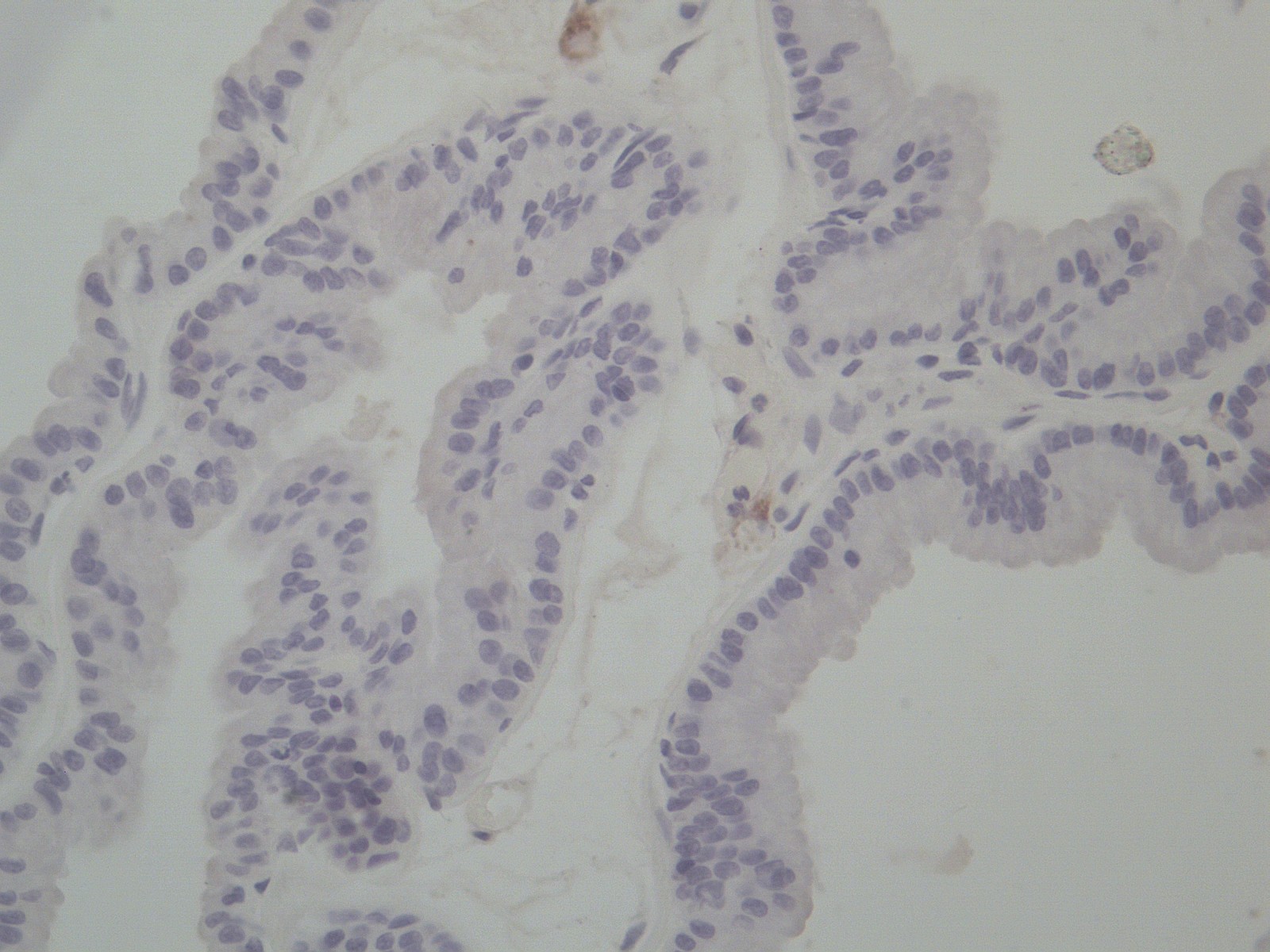

Supplement: Supplementary Materials — Five original photos in Figure 5 and the description of their applications in the study. [file 7312938.f1.zip › Yin Zhi Huang Soup group/5.jpg]
